# Clinical outcomes of catheter ablation for atrial fibrillation, atrial flutter, and atrial tachycardia in wild-type transthyretin amyloid cardiomyopathy: a proposed treatment strategy for catheter ablation in each arrhythmia

**DOI:** 10.1093/europace/euae155

**Published:** 2024-06-27

**Authors:** Hisanori Kanazawa, Seiji Takashio, Tadashi Hoshiyama, Miwa Ito, Shozo Kaneko, Takuya Kiyama, Yusei Kawahara, Hitoshi Sumi, Yuichiro Tsuruta, Naoto Kuyama, Kyoko Hirakawa, Masanobu Ishii, Noriaki Tabata, Kenshi Yamanaga, Koichiro Fujisue, Shinsuke Hanatani, Daisuke Sueta, Yuichiro Arima, Satoshi Araki, Yasushi Matsuzawa, Hiroki Usuku, Taishi Nakamura, Eiichiro Yamamoto, Hirofumi Soejima, Kenichi Matsushita, Kenichi Tsujita

**Affiliations:** Department of Cardiovascular Medicine, Graduate School of Medical Sciences, Kumamoto University, 1-1-1 Honjo, Chuo-ku, Kumamoto 860-8556, Japan; Department of Cardiac Arrhythmias, Kumamoto University, 1-1-1 Honjo, Chuo-ku, Kumamoto 860-8556, Japan; Department of Cardiovascular Medicine, Graduate School of Medical Sciences, Kumamoto University, 1-1-1 Honjo, Chuo-ku, Kumamoto 860-8556, Japan; Department of Cardiovascular Medicine, Graduate School of Medical Sciences, Kumamoto University, 1-1-1 Honjo, Chuo-ku, Kumamoto 860-8556, Japan; Department of Cardiovascular Medicine, Graduate School of Medical Sciences, Kumamoto University, 1-1-1 Honjo, Chuo-ku, Kumamoto 860-8556, Japan; Department of Cardiovascular Medicine, Graduate School of Medical Sciences, Kumamoto University, 1-1-1 Honjo, Chuo-ku, Kumamoto 860-8556, Japan; Department of Cardiovascular Medicine, Graduate School of Medical Sciences, Kumamoto University, 1-1-1 Honjo, Chuo-ku, Kumamoto 860-8556, Japan; Department of Cardiovascular Medicine, Graduate School of Medical Sciences, Kumamoto University, 1-1-1 Honjo, Chuo-ku, Kumamoto 860-8556, Japan; Department of Cardiovascular Medicine, Graduate School of Medical Sciences, Kumamoto University, 1-1-1 Honjo, Chuo-ku, Kumamoto 860-8556, Japan; Department of Cardiovascular Medicine, Graduate School of Medical Sciences, Kumamoto University, 1-1-1 Honjo, Chuo-ku, Kumamoto 860-8556, Japan; Department of Cardiovascular Medicine, Graduate School of Medical Sciences, Kumamoto University, 1-1-1 Honjo, Chuo-ku, Kumamoto 860-8556, Japan; Department of Cardiovascular Medicine, Graduate School of Medical Sciences, Kumamoto University, 1-1-1 Honjo, Chuo-ku, Kumamoto 860-8556, Japan; Department of Cardiovascular Medicine, Graduate School of Medical Sciences, Kumamoto University, 1-1-1 Honjo, Chuo-ku, Kumamoto 860-8556, Japan; Department of Cardiovascular Medicine, Graduate School of Medical Sciences, Kumamoto University, 1-1-1 Honjo, Chuo-ku, Kumamoto 860-8556, Japan; Department of Cardiovascular Medicine, Graduate School of Medical Sciences, Kumamoto University, 1-1-1 Honjo, Chuo-ku, Kumamoto 860-8556, Japan; Department of Cardiovascular Medicine, Graduate School of Medical Sciences, Kumamoto University, 1-1-1 Honjo, Chuo-ku, Kumamoto 860-8556, Japan; Department of Cardiovascular Medicine, Graduate School of Medical Sciences, Kumamoto University, 1-1-1 Honjo, Chuo-ku, Kumamoto 860-8556, Japan; Department of Cardiovascular Medicine, Graduate School of Medical Sciences, Kumamoto University, 1-1-1 Honjo, Chuo-ku, Kumamoto 860-8556, Japan; Department of Cardiovascular Medicine, Graduate School of Medical Sciences, Kumamoto University, 1-1-1 Honjo, Chuo-ku, Kumamoto 860-8556, Japan; Department of Cardiovascular Medicine, Graduate School of Medical Sciences, Kumamoto University, 1-1-1 Honjo, Chuo-ku, Kumamoto 860-8556, Japan; Department of Cardiovascular Medicine, Graduate School of Medical Sciences, Kumamoto University, 1-1-1 Honjo, Chuo-ku, Kumamoto 860-8556, Japan; Department of Cardiovascular Medicine, Graduate School of Medical Sciences, Kumamoto University, 1-1-1 Honjo, Chuo-ku, Kumamoto 860-8556, Japan; Department of Cardiovascular Medicine, Graduate School of Medical Sciences, Kumamoto University, 1-1-1 Honjo, Chuo-ku, Kumamoto 860-8556, Japan; Department of Cardiovascular Medicine, Graduate School of Medical Sciences, Kumamoto University, 1-1-1 Honjo, Chuo-ku, Kumamoto 860-8556, Japan; Department of Cardiovascular Medicine, Graduate School of Medical Sciences, Kumamoto University, 1-1-1 Honjo, Chuo-ku, Kumamoto 860-8556, Japan; Department of Cardiovascular Medicine, Graduate School of Medical Sciences, Kumamoto University, 1-1-1 Honjo, Chuo-ku, Kumamoto 860-8556, Japan; Department of Cardiovascular Medicine, Graduate School of Medical Sciences, Kumamoto University, 1-1-1 Honjo, Chuo-ku, Kumamoto 860-8556, Japan

**Keywords:** Wild-type transthyretin amyloid cardiomyopathy, Catheter ablation, Atrial fibrillation, Atrial flutter, Atrial tachycardia

## Abstract

**Aims:**

Wild-type transthyretin amyloid cardiomyopathy (ATTRwt-CM) is often accompanied by atrial fibrillation (AF), atrial flutter (AFL), and atrial tachycardia (AT), which are difficult to control because beta-blockers and antiarrhythmic drugs can worsen heart failure (HF). This study aimed to investigate the outcomes of catheter ablation (CA) for AF/AFL/AT in patients with ATTRwt-CM and propose a treatment strategy for CA.

**Methods and results:**

A cohort study was conducted on 233 patients diagnosed with ATTRwt-CM, including 54 who underwent CA for AF/AFL/AT. The background of each arrhythmia and the details of the CA and its outcomes were investigated. The recurrence-free rate of AF/AFL/AT overall in ATTRwt-CM patients with multiple CA was 70.1% at 1-year, 57.6% at 2-year, and 44.0% at 5-year follow-up, but CA significantly reduced all-cause mortality [hazard ratio (HR): 0.342, 95% confidence interval (CI): 0.133–0.876, *P* = 0.025], cardiovascular mortality (HR: 0.378, 95% CI: 0.146–0.981, *P* = 0.045), and HF hospitalization (HR: 0.488, 95% CI: 0.269–0.889, *P* = 0.019) compared with those without CA. There was no recurrence of the cavotricuspid isthmus (CTI)–dependent AFL, non-CTI–dependent simple AFL terminated by one linear ablation, and focal AT originating from the atrioventricular (AV) annulus or crista terminalis eventually. Twelve of 13 patients with paroxysmal AF and 27 of 29 patients with persistent AF did not have recurrence as AF. However, all three patients with non-CTI–dependent complex AFL not terminated by a single linear ablation and 10 of 13 cases with focal AT or multiple focal ATs originating beyond the AV annulus or crista terminalis recurred even after multiple CA.

**Conclusion:**

The outcomes of CA for ATTRwt-CM were acceptable, except for multiple focal AT and complex AFL. Catheter ablation may be aggressively considered as a treatment strategy with the expectation of improving mortality and hospitalization for HF.

## Graphical Abstract

**Graphical Abstract:**
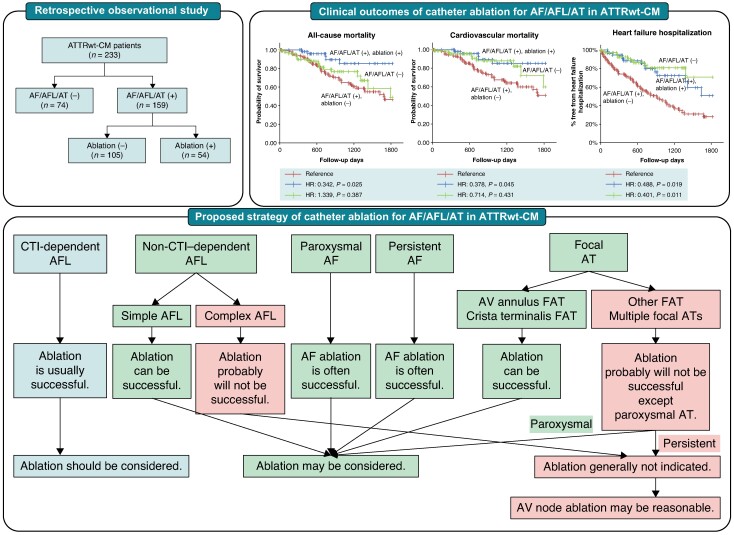

**What’s new?**

- In wild-type transthyretin amyloid cardiomyopathy, catheter ablation (CA) for atrial fibrillation (AF)/atrial flutter (AFL)/atrial tachycardia (AT) significantly reduced all-cause mortality, cardiovascular mortality, and heart failure hospitalization compared with those without CA.
- Especially, CA is effective for cavotricuspid isthmus (CTI)–dependent AFL, non-CTI–dependent simple AFL terminated by a single line, paroxysmal/persistent AF, and focal AT originated from the atrioventricular annulus and crista terminalis.
- Catheter ablation is not useful for focal AT and multiple focal ATs originating from other sites, and non-CTI–dependent complex AFL not terminated by a single line, and recurrence is especially dependent on the presence of AT.
- However, the outcomes of CA for AF/AFL/AT in patients with wild-type transthyretin amyloid cardiomyopathy are not necessarily poor, and CA may be a treatment option depending on the patient’s condition to improve prognosis and hospitalization for heart failure.

## Introduction

Since the recent report demonstrated that technetium 99m pyrophosphate (^99m^Tc-PYP) scintigraphy is highly sensitive and positive in cardiac amyloidosis caused by transthyretin (TTR), it has become clear that amyloidosis, especially wild-type TTR amyloidosis (ATTRwt), which had been considered a rare disease, is more common than previously thought and is encountered relatively frequently in daily practice.^1,2^ Furthermore, in ATTRwt cardiomyopathy (ATTRwt-CM), atrial fibrillation (AF), atrial flutter (AFL), and atrial tachycardia (AT) are reported to occur as frequently as 27–67%^3–7^ following the direct effect of TTR amyloid deposition in the atrium and atrial overload due to impaired ventricular dilatation and increased ventricular filling pressure and are associated with the development of heart failure (HF).^8^ However, because cardiac amyloidosis tends to cause a decrease in stroke volume and blood pressure based on the restrictive disorder, HF drugs including β-blockers, heart rate control drugs such as digitalis and verapamil, and even antiarrhythmic drugs should not be administered in principle because of the risk of a negative inotropic effect.^9^

Therefore, when AF, AFL, or AT is accompanied by ATTRwt-CM, it is often difficult to achieve sinus rhythm or control the heart rate, resulting in worsening HF. Meanwhile, catheter ablation (CA) is considered a non-pharmacologic treatment for AF/AFL/AT associated with ATTRwt-CM, but we encountered a case of multiple focal ATs that were extremely intractable for CA.^10^ A total of eight multiple focal ATs were ablated in two sessions; however, they recurred easily, and eventually, atrioventricular (AV) node ablation was needed to be followed by cardiac resynchronization therapy defibrillator implantation. Nevertheless, some patients with ATTRwt-CM have been successfully treated with CA depending on the type of arrhythmia.

In this study, we investigated the characteristics of AF/AFL/AT associated with ATTRwt-CM and the treatment and outcomes of CA to identify patients for whom CA treatment was effective or ineffective.

## Methods

### Study population

**Figure 1:**
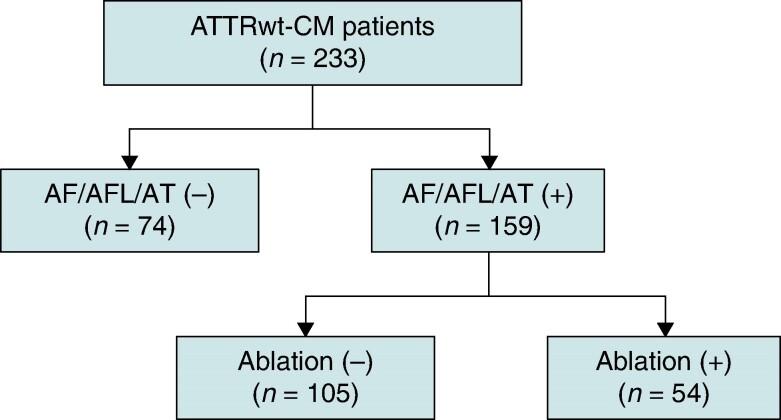
Study flow chart 1. AF, atrial fibrillation; AFL, atrial flutter; AT, atrial tachycardia; ATTRwt-CM, wild-type transthyretin amyloid cardiomyopathy.

**Figure 2:**
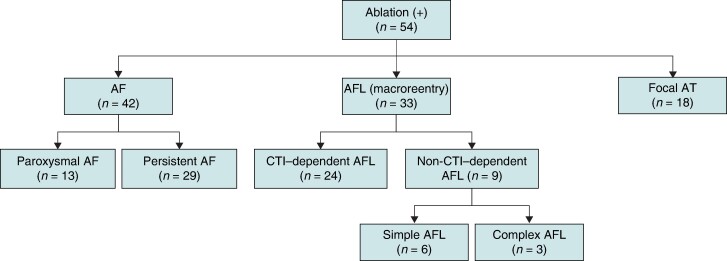
Study flow chart 2 (details of arrhythmias). AF, atrial fibrillation; AFL, atrial flutter; AT, atrial tachycardia; CTI, cavotricuspid isthmus.

This cohort study included 233 consecutive patients diagnosed with ATTRwt-CM at the Kumamoto University Hospital. Wild-type transthyretin amyloid cardiomyopathy was diagnosed using ^99m^Tc-PYP scintigraphy, tissue biopsy, monoclonal protein evaluation, and TTR genetic screening, as described in previous reports.^7,11^ Among a total of 233 ATTRwt-CM patients, AF/AFL/AT was present in 159 patients (68%), of whom 54 underwent CA (*Figure 1*, and *Tables 1* and *2*). The total number exceeded 54 because some patients had multiple arrhythmias in a single patient; however, CA was performed in 42 patients for AF, 33 for AFL as a macroreentry, and 18 for AT as a focal pattern rather than a macroreentry, which were finally diagnosed according to the intraoperative findings during CA (*Figure 2*). There were 13 patients with paroxysmal AF and 29 with persistent AF. Atrial flutter was divided into 24 cases of cavotricuspid isthmus (CTI)–dependent AFL and 9 cases of other non-CTI–dependent AFL. Furthermore, non-CTI–dependent AFLs were divided into two groups: those in which the circuit could be identified and the AFL was terminated with a single linear ablation (simple AFL) and those in which the circuit was difficult to identify, multiple slow conduction zones (SCZs) were present, and multiple line creation was needed to terminate the tachycardia (complex AFL; *Figure 2*).

**Table 1:** Baseline characteristic in ATTRwt-CM patients with and without AF/AFL/AT

Variable	AF/AFL/AT (−)	AF/AFL/AT (+)	AF/AFL/AT (+)	*P* value
		Ablation (−)	Ablation (+)	
	*n* = 74 (32%)	*n* = 105 (45%)	*n* = 54 (23%)	
Age (years)	79 (74–84)	79 (74–84)	75 (70–78)	<0.001
Male, *n* (%)	62 (84)	90 (86)	48 (89)	0.715
Height (cm)	161.0 (154.0–166.1)	162.0 (154.8–167.3)	164.5 (158.5–168.4)	0.108
Weight (kg)	59.7 (48.9–64.4)	58.2 (52.0–65.1)	61.2 (53.1–68.1)	0.295
^99m^Tc-PYP, *n* (%)	73 (99)	102 (97)	53 (98)	0.340
Cardiac MRI, *n* (%)	39 (53)	54 (51)	37 (69)	0.098
Biopsy, *n* (%)	45 (61)	77 (73)	41 (76)	0.109
NYHA				<0.001
Ⅰ, *n* (%)	20 (27)	6 (6)	10 (18)	
Ⅱ, *n* (%)	32 (43)	50 (47)	28 (52)	
Ⅲ, *n* (%)	21 (29)	47 (45)	16 (30)	
Ⅳ, *n* (%)	1 (1)	2 (2)	0 (0)	
Arrhythmia				
Paroxysmal AF, *n* (%)	0 (0)	13 (12)	12 (22)	<0.001
Persistent AF, *n* (%)	0 (0)	73 (70)	31 (57)	<0.001
AFL, *n* (%)	0 (0)	24 (23)	26 (48)	<0.001
AT, *n* (%)	0 (0)	4 (4)	21 (39)	<0.001
Hypertension, *n* (%)	41 (55)	56 (53)	28 (52)	0.920
Systolic blood pressure (mmHg)	123 (113–132)	113 (104–132)	115 (105–125)	0.022
Diastolic blood pressure (mmHg)	71 (63–80)	67 (61–77)	68 (63–81)	0.297
Diabetes mellitus, *n* (%)	14 (19)	27 (26)	11 (20)	0.526
Dyslipidaemia, *n* (%)	25 (34)	29 (28)	18 (33)	0.617
Old myocardial infarction, *n* (%)	2 (3)	6 (6)	0 (0)	0.159
History of PCI/CABG, *n* (%)	6 (8)	9 (9)	3 (6)	0.788
hs-cTnT (ng/mL)	0.044 (0.028–0.069)	0.064 (0.047–0.099)	0.043 (0.027–0.066)	<0.001
BNP (pg/mL)	178.0 (86.3–335.6)	289.0 (185.8–471.4)	187.4 (108.6–348.0)	<0.001
eGFR (mL/min/1.73 m^2^)	53.99 ± 15.21	48.51 ± 15.10	55.46 ± 13.85	0.007
Echocardiographic parameters				
LVDd (mm)	40.7 (37.1–44.5)	40.2 (37.2–45.5)	42.7 (38.1–45.0)	0.670
LVDs (mm)	29.7 (25.8–33.6)	30.7 (26.8–35.7)	32.4 (27.9–36.1)	0.114
IVSTd (mm)	14.7 (13.1–16.6)	15.6 (13.8–17.5)	15.1 (13.6–16.7)	0.065
PLVWd (mm)	15.11 ± 3.01	15.81 ± 2.87	15.79 ± 2.40	0.223
LVEF (%)	54.1 (46.4–60.1)	51.5 (42.1–60.1)	53.2 (42.5–58.0)	0.239
LADs (mm)	38.6 ± 5.7	44.1 ± 7.8	41.9 ± 5.5	<0.001
*E*/*e*′	18.8 (11.6–24.2)	20.4 (16.6–25.8)	18.7 (12.5–22.2)	0.157
Dct (ms)	184.5 (147.9–226.9)	164.0 (139.5–200.5)	157.0 (127.7–218.7)	0.049
Medications				
RAS inhibitors, *n* (%)	29 (39)	51 (49)	25 (46)	0.452
MRAs, *n* (%)	16 (22)	41 (39)	12 (22)	0.017
β-Blockers, *n* (%)	9 (12)	38 (36)	18 (33)	0.001
Loop or thiazide diuretics, *n* (%)	35 (47)	89 (85)	35 (65)	<0.001
Amiodarone, *n* (%)	1 (1)	1 (1)	5 (9)	0.419

Data are presented as mean ± standard deviation, median value with interquartile range, or frequencies and percentages (%).

^99m^Tc-PYP, technetium 99m pyrophosphate cardiac scintigraphy; AF, atrial fibrillation; AFL, atrial flutter; AT, atrial tachycardia; ATTRwt-CM, wild-type transthyretin amyloid cardiomyopathy; BNP, brain natriuretic peptide; CABG, coronary artery bypass grafting; Dct, deceleration time; eGFR, estimated glomerular filtration ratio; hs-cTnT, high-sensitivity cardiac troponin T; IVSTd, interventricular septal wall thickness at end-diastole; LADs, left atrial end-systolic diameter; LVDd, left ventricular end-diastolic diameter; LVDs, left ventricular end-systolic diameter; LVEF, left ventricular ejection fraction; MRA, mineralocorticoid receptor antagonist; MRI, magnetic resonance imaging; NYHA, New York Heart Association; PCI, percutaneous coronary intervention; PLVWd, posterior left ventricular wall thickness at end-diastole; RAS, renin–angiotensin system.

**Table 2:** Adjusted hazard ratios for all-cause mortality, cardiovascular mortality, and heart failure hospitalization due to the occurrence of AF/AFL/AT or ablation for it in patients with ATTRwt-CM

ATTRwt-CM (*n* = 233)	AF/AFL/AT (+), ablation (−), *n* = 105 (45%)	AF/AFL/AT (+), ablation (+), *n* = 54 (23%)	AF/AFL/AT (−), *n* = 74 (32%)	Adjusted HR^a^ (95% CI)		Adjusted HR^a^ (95% CI)	
				Catheter ablation	*P* value	AF/AFL/AT (−)	*P* value
All-cause mortality, *n* (%)	40 (38)	5 (9)	17 (23)	0.342 (0.133–0.876)	0.025	1.339 (0.691–2.593)	0.387
Cardiovascular mortality, *n* (%)	35 (33)	5 (9)	9 (12)	0.378 (0.146–0.981)	0.045	0.714 (0.308–1.652)	0.431
Heart failure hospitalization, *n* (%)	60 (57)	14 (26)	11 (15)	0.488 (0.269–0.889)	0.019	0.401 (0.198–0.814)	0.011

CI, confidence interval; other abbreviations as in *Table 1*.

^a^Hazard ratios (HRs) are adjusted for non-internal correlated factors, i.e. NYHA, LADs, Dct, β-blockers, and loop or thiazide diuretics, and adjusted HRs are shown in comparison with those who developed AF/AFL/AT but did not undergo ablation as the reference. The correlation coefficients between NYHA and age, hs-cTnT, BNP, and eGFR were 0.430 (*P* < 0.001), 0.439 (*P* < 0.001), 0.476 (*P* < 0.001), and −0.348 (*P* < 0.001), respectively. The correlation coefficient between loop or thiazide diuretics and mineralocorticoid receptor antagonist was 0.301 (*P* < 0.001).

The diagnosis of AT or AFL in cases who have not undergone ablation or who have recurred post-operatively but not undergone reablation was made by the presence of a flutter wave [sawtooth flutter wave or continual electrical activity (lack of an isoelectric interval between flutter waves)] on a 12-lead electrocardiogram (ECG) or the rate of the P-wave on a 12-lead ECG or atrial wave on an implantable electrical device (>250 b.p.m. for AFL and <250 b.p.m. for AT).^12^ The type of AF, paroxysmal or persistent, was determined according to the 2017 expert consensus.^13^

This study was conducted in accordance with the principles of the Declaration of Helsinki. This study was approved by the Institutional Review Board and Ethics Committee of Kumamoto University (approval no. 1590). The requirement for informed consent was waived because the risk of this cohort study was low and direct consent could not be obtained from all subjects. Therefore, we widely advertised the study protocol at the Kumamoto University Hospital and on our website (http://www.kumadai-junnai.com), providing patients with the opportunity to opt-out.

### Indication and strategy of catheter ablation

The CA was performed between May 2017 and June 2022, except for five patients with CTI-dependent AFL [August 2009, May 2010, May 2012, May 2014, and February 2016 (patient nos. 6, 9, 16, 20, and 21 in *Table 3*)], zero patient with non-CTI-dependent AFL (*Table 4*), four patients with AF [January 2014 and February 2016 (patient nos. 1 and 11 in *Table 5*) and May 2015 and October 2015 (patient nos. 1 and 15 in *Table 6*)], and two patients with AT [May 2014 and September 2016 (patient nos. 3 and 4 in *Table 7*)]. Screening for AF/AFL/AT was performed by ECG at physical examination, during symptoms, at the time of diagnosis of ATTRwt-CM, or every year following. The indications for CA for AF/AFL/AT were considered according to guidelines,^13–15^ with no special criteria established because of ATTRwt-CM, and CA was performed in symptomatic AF/AFL/AT patients with palpitations or signs of HF as described in the Supplementary material. Furthermore, CA was aggressively performed in asymptomatic AF/AFL/AT patients whose arrhythmia was paroxysmal or persistent for less than 1 year, with the expectation of improved exercise tolerance, B-type natriuretic peptide (BNP), and quality of life as good as symptomatic patients.^16–18^ However, CA was not performed in patients with chronic sustaining arrhythmia prior to ATTRwt-CM diagnosis or no symptoms at all and no desire for ablation.

**Table 3:** Characteristic of ATTRwt-CM patient who underwent catheter ablation for CTI-dependent AFL

Patient no.	Age/sex (years)		Recurrence of CTI-dependent AFL	Paroxysmal/persistent	MRI-ECV (%)	Native T_1_ (ms)	PyP	Biopsy	hs-cTnT (ng/mL)	eGFR (mL/min /1.73 m^2^)	BNP (pg/mL)	LVDd (mm)	LVDs (mm)	IVSTd (mm)	PLVWd (mm)	LVEF (%)	LADs (mm)	*E*/*e*′	Ablation lesions	Sustained non-CTI–dependent AFL/FAT remaining or induced at end of the session	Recurrence of AF/non-CTI–dependent AFL/FAT
1	69	M	No	Persistent	69.8	1472	+	+	0.0484	41.8	311.7	41.4	30.0	17.2	15.6	55.0	42.2	18.2	CTI, AFL	Simple AFL	Yes/AFL (reABL)
2	67	M	No	Paroxysmal	80.0	1504	+	+	0.0281	87.1	101.5	47.3	33.0	13.1	12.2	51.7	37.3	14.0	CTI, AT	FAT	Yes/FAT (reABL)
3	63	M	No	Persistent	69.0	1451	+	+	0.0717	54.0	589.7	41.1	32.8	14.5	15.3	55.0	46.5	18.7	CTI, AT	Multiple FATs	Yes/FAT (ECG)
4	80	M	No	Persistent	69.3	1431	+	+	0.0644	53.0	77.1	39.3	30.0	13.5	16.3	51.6	40.9	20.1	CTI, AT	Multiple FATs	Yes/FAT (reABL)
5	71	M	No	Persistent	61.1	1457	+	+	0.0789	50.0	281.2	40.2	34.6	14.2	16.6	33.4	36.2	18.3	CTI, AFL	Complex AFL	Yes/AFL (reABL)
																					
6	79	M	No	Paroxysmal	N/A	N/A	+	+	0.1015	68.1	97.1	32.0	20.0	15.0	16.0	64.7	56.1	19.5	CTI	−	Yes/AF
7	78	M	No	Persistent	32.4	1280	+	N/A	0.0184	43.0	209.3	30.7	19.6	14.3	14.6	59.7	38.3	13.0	CTI	−	Yes/AF
8	83	M	No	Persistent	N/A	N/A	+	N/A	0.0723	40.0	726.7	36.0	26.0	18.0	18.0	52.0	41.0	31.1	CTI	−	Yes/AF
9	73	M	No	Persistent	58.0	1449	+	+	0.0200	65.1	149.7	41.0	27.0	13.0	14.0	60.0	36.0	11.2	CTI	−	No
10	64	M	No	Persistent	N/A	N/A	+	+	0.0272	48.0	30.8	35.2	34.9	17.6	18.8	33.7	38.8	11.7	CTI	−	No
11	82	M	No	Paroxysmal	39.3	1347	+	N/A	0.0374	35.0	89.6	39.5	28.2	15.6	18.5	53.7	42.6	18.3	CTI	−	No
12	59	M	No	Paroxysmal	51.5	1413	+	+	0.0194	57.0	68.5	42.8	25.9	13.6	14.1	72.0	44.7	17.3	CTI	−	No
13	73	M	No	Persistent	77.0	1524	+	+	0.0227	54.0	126.1	38.6	29.1	16.9	16.9	49.7	35.9	23.3	CTI	−	No
14	93	F	No	Persistent	N/A	N/A	+	+	0.0700	27.0	205.5	31.9	16.8	11.4	12.3	48.2	43.4	14.1	CTI	−	No
																					
15	79	M	No	Persistent	N/A	N/A	+	N/A	0.0200	64.0	266.0	35.2	24.8	19.2	19.2	58.6	45.3	18.1	CTI	N/A	Yes/AF
16	74	M	No	Persistent	N/A	N/A	+	+	0.0212	55.0	212.1	43.3	24.6	13.8	11.3	62.1	43.2	11.3	CTI	N/A	Yes/AF, AFL, FAT (reABL)
17	82	M	No	Persistent	N/A	N/A	+	+	0.0554	54.2	532.0	51.0	45.0	9.0	10.0	30.0	45.0	9.5	CTI	N/A	Yes/FAT (reABL)
18	74	M	No	N/A	46.3	1397	+	+	0.0202	65.0	127.2	53.7	40.8	13.0	11.0	47.0	45.0	12.8	CTI	N/A	Yes/AF
19	66	M	No	Persistent	68.2	1414	+	+	0.0405	53.0	150.4	50.7	34.6	11.8	11.2	59.4	46.2	12.3	CTI	N/A	Yes/AFL, FAT (reABL)
20	77	M	No	Persistent	N/A	N/A	+	N/A	0.0612	64.0	98.8	39.6	35.5	19.4	18.7	42.0	38.8	23.3	CTI	N/A	No
21	72	M	No	N/A	N/A	N/A	+	+	0.0860	57.0	98.9	49.8	37.8	12.4	12.5	48.0	44.4	33.1	CTI	N/A	No
22	81	M	No	Persistent	N/A	N/A	+	N/A	0.0401	40.0	41.0	37.3	28.2	13.6	14.7	47.9	32.2	6.8	CTI	N/A	No
23	80	M	No	Persistent	69.3	1382	+	N/A	0.0592	52.0	378.2	41.2	27.1	17.5	15.9	63.1	48.0	16.0	CTI	N/A	No
24	75	M	No	Persistent	N/A	N/A	+	+	0.0695	32.0	266.4	32.7	24.2	15.4	18.1	41.1	31.6	34.8	CTI	N/A	No

CTI, cavotricuspid isthmus; ECG, electrocardiogram; ECV, extracellular volume fraction; FAT, focal atrial tachycardia; N/A, not available; PyP, technetium 99m pyrophosphate cardiac scintigraphy; reABL, reablation; other abbreviations as in *Table 1*.

**Table 4:** Characteristic of ATTRwt-CM patient who underwent catheter ablation for non-CTI–dependent AFL (macroreentrant AFL)

Patient no.	Age/sex (years)		Recurrence of AF/non-CTI–dependent AFL/FAT	Paroxysmal /persistent	MRI-ECV (%)	Native T_1_ (ms)	PyP	Biopsy	hs-cTnT (ng/mL)	eGFR (mL/min /1.73m^2^)	BNP (pg/mL)	LVDd (mm)	LVDs (mm)	IVSTd (mm)	PLVWd (mm)	LVEF (%)	LADs (mm)	E/e′	Ablation lesions	Sustained non-CTI–dependent AFL/FAT remaining or induced at the end of the session
AFL terminated by a single line (simple AFL)																				
1	69 M		Yes/AFL (reABL)	Persistent	69.8	1472	+	+	0.0484	41.8	311.7	41.4	30.0	17.2	15.6	55.0	42.2	18.2	Mitral IS	N/A
	69 M		No	Persistent	69.8	1472	+	+	0.0510	34.0	520.1	47.8	40.4	17.6	15.9	35.0	46.1	15.0	Mitral IS	N/A
2	81 M		No	Persistent	N/A	N/A	+	+	0.1390	31.0	929.5	51.7	39.8	13.4	13.3	36.9	33.1	14.9	Mitral IS	−
3	83 M		No	Persistent	N/A	N/A	+	+	0.1638	46.0	539.8	49.6	41.2	12.7	13.1	41.1	44.1	9.1	LA anterior	N/A
4	82 M		No	Paroxysmal	39.3	1347	+	N/A	0.0374	35.0	89.6	39.5	28.2	15.6	18.5	53.7	42.6	18.3	LA anterior, SVC	−
5	66 M		No	Paroxysmal	68.2	1414	+	+	0.0586	51.0	269.6	42.6	33.1	13.2	13.5	47.0	47.8	12.3	Mitral IS	N/A
6	73 M		No	Persistent	77.0	1524	+	+	0.0227	54.0	126.1	38.6	29.1	16.9	16.9	49.7	35.9	23.3	LA anterior	−

AFL not terminated by a single line (complex AFL)																				
7	84 M		Yes/AFL (ECG)	Persistent	47.0	1395	+	N/A	0.0612	55.0	186.0	34.0	25.9	15.2	13.1	63.0	27.1	9.3	LA posterior, LA posteroinferior LA floor	Complex AFL
8	71 M		Yes/AFL (reABL)	Persistent	61.1	1457	+	+	0.0789	50.0	281.2	40.2	34.6	14.2	16.6	33.4	36.2	18.3	LA roof, SVC Bachmann bundle	−
	71 M		Yes/AFL (ECG)	Persistent	61.1	1457	+	+	0.0730	41.0	381.2	42.7	35.0	14.3	15.2	32.5	36.2	22.8	RA posterior, LA posterior roof	−
9	78 M		Yes/AFL (ECG)	Persistent	65.4	1381	+	+	0.0686	48.0	582.6	41.6	34.6	13.8	14.4	40.6	40.1	14.5	RPV carina, LA floor, LA roof, LA septum	N/A

IS, isthmus; LA, left atrium; RA, right atrium; RPV, right pulmonary vein; SVC, superior vena cava; other abbreviations as in *Tables 1* and *3*.

**Table 5:** Characteristic of ATTRwt-CM patient who underwent catheter ablation for paroxysmal AF

Patient no.	Age/sex (years)		Recurrence of AF	Recurrence of non-CTI–dependent AFL/FAT	MRI-ECV (%)	Native T_1_ (ms)	PyP	Biopsy	hs-cTnT (ng/mL)	eGFR (mL/min /1.73 m^2^)	BNP (pg/mL)	LVDd (mm)	LVDs (mm)	IVSTd (mm)	PLVWd (mm)	LVEF (%)	LADs (mm)	*E*/*e*′	Ablation lesions	Sustained non-CTI–dependent AFL/FAT remaining or induced during session	Recurrence arrhythmia
1	72	M	Yes	No	47.0	1425	+	+	0.0408	62.0	179.4	43.5	35.3	15.5	14.7	44.5	37.2	9.5	PVI	N/A	AF
2	78	M	Yes	No	32.4	1280	+	N/A	0.0184	43.0	209.3	30.7	19.6	14.3	14.6	59.7	38.3	13.0	PVI	−	AF
	79	M	No	No	32.4	1280	+	N/A	0.0146	49.0	94.2	41.3	21.8	12.5	11.6	66.3	41.6	15.4	RPVI	−	
																					
3	67	M	No	Yes	80.0	1504	+	+	0.0281	87.1	101.5	47.3	33.0	13.1	12.2	51.7	37.3	14.0	PVI, AT	FAT	FAT (reABL)
4	73	M	No	Yes	N/A	N/A	+	+	0.0418	49.2	185.8	40.9	34.9	13.1	13.5	43.3	42.8	20.6	PVI, AT	multiple FATs	FAT (reABL)
5	71	M	No	Yes	45.5	1300	+	+	0.0468	37.0	401.7	43.4	31.5	12.4	12.2	54.4	28.4	21.6	PVI, AT	FAT	AT (ECG)
6	80	F	No	Yes	48.9	1477	+	+	0.0294	37.0	95.5	46.7	34.7	15.6	14.6	53.9	37.8	7.9	PVI	FAT	AT (ECG)
																					
7	59	M	No	No	51.5	1413	+	+	0.0194	57.0	68.5	42.8	25.9	13.6	14.1	72.0	44.7	17.3	PVI	−	
8	82	M	No	No	39.3	1347	+	N/A	0.0374	35.0	89.6	39.5	28.2	15.6	18.5	53.7	42.6	18.3	PVI	−	
9	79	M	No	No	58.3	1472	+	+	0.0610	39.0	256.1	44.8	31.1	17.1	16.9	58.1	51.5	41.2	PVI, non-PV foci	−	
10	93	F	No	No	N/A	N/A	+	+	0.0700	27.0	205.5	31.9	16.8	11.4	12.3	48.2	43.4	14.1	PVI	−	
11	72	M	No	No	N/A	N/A	+	+	0.0860	57.0	98.9	49.8	37.8	12.4	12.5	48.0	44.4	33.1	PVI	N/A	
12	75	M	No	No	37.0	1368	+	+	0.0224	57.0	31.4	48.9	31.6	11.5	12.5	64.6	38.5	11.6	PVI	N/A	
13	80	M	No	No	69.3	1382	+	N/A	0.0592	52.0	378.2	41.2	27.1	17.5	15.9	63.1	48.0	16.0	PVI	N/A	

PV, pulmonary vein; PVI, pulmonary vein isolation; RPVI, right pulmonary vein isolation; other abbreviations as in *Tables 1* and *3*.

**Table 6:** Characteristic of ATTRwt-CM patient who underwent catheter ablation for persistent AF

Patient no.	Age/sex (years)		Recurrence of AF	Recurrence of non-CTI–dependent AFL/FAT	MRI-ECV (%)	Native T_1_ (ms)	PyP	Biopsy	hs-cTnT (ng/mL)	eGFR (mL/min /1.73 m^2^)	BNP (pg/mL)	LVDd (mm)	LVDs (mm)	IVSTd (mm)	PLVWd (mm)	LVEF (%)	LADs (mm)	*E*/*e*′	Ablation lesions	Sustained non-CTI–dependent AFL/FAT remaining or induced during session	Recurrence arrhythmia
1	70	M	Yes	No	N/A	N/A	+	+	0.0740	76.1	92.4	40.1	23.2	14.2	12.9	70.9	54.6	11.8	PVI	N/A	AF
2	74	M	Yes	No	46.3	1397	+	+	0.0202	65.0	127.2	53.7	40.8	13.0	11.0	47.0	45.0	12.8	PVI	N/A	AF
	76	M	No	No	46.3	1397	+	+	0.0222	59.0	394.8	53.5	44.4	13.5	13.2	61.9	35.7	11.7	Box	N/A	
3	78	M	Yes	No	47.0	1370	+	+	0.0208	56	101.2	43.4	30.4	13.4	12.6	59.3	43.9	22.1	PVI, box	N/A	AF
																					
4	79	M	No	Yes	N/A	N/A	+	+	0.0700	53.0	595.8	50.4	36.6	13.9	12.1	53.4	49.0	10.6	PVI, AFL (unsuccessful)	AFL	AFL (reABL)
5	69	M	No	Yes	69.8	1472	+	+	0.0484	41.8	311.7	41.4	30.0	17.2	15.6	55.0	42.2	18.2	PVI, box, AFL	AFL	AFL (reABL)
6	77	F	No	Yes	N/A	N/A	+	+	0.0330	38.3	54.5	40.7	28.0	14.6	15.0	50.0	37.3	19.4	PVI, box, AT	FAT	AT/AFL (ECG)
7	83	M	No	Yes	N/A	N/A	+	+	0.1860	51.0	166.0	45.0	41.0	12.1	12.2	34.9	42.8	9.7	PVI, AT	Multiple FATs	AT (ECG)
8	78	M	No	Yes	89.0	1444	+	+	0.1215	66.0	496.5	46.2	40.5	14.1	16.1	35.9	43.9	11.1	PVI, AT	Multiple FATs	FAT (reABL)
9	78	F	No	Yes	N/A	N/A	+	+	0.0132	46.0	266.2	46.1	36.8	10.0	9.5	45.0	43.5	27.6	PVI, AT	FAT	AT/AFL (ECG)
10	74	M	No	Yes	83.0	1416	N/A	+	0.0797	63.0	231.7	43.7	38.5	15.3	15.2	34.4	46.6	17.5	PVI	FAT	AT (ECG)
11	78	M	No	Yes	65.4	1381	+	+	0.0686	48.0	582.6	41.6	34.6	13.8	14.4	40.6	40.1	14.5	PVI, AFL	Complex AFL	AT/AFL (ECG)
12	69	M	No	Yes	61.1	1457	+	+	0.0956	61.1	667.7	43.5	32.8	12.1	12.1	53.0	41.5	33.6	PVI, box	−	complex AFL (reABL)
13	83	M	No	Yes	47.4	1395	+	N/A	0.0127	76.0	219.9	40.6	25.1	13.4	12.5	68.9	42.9	12.3	PVI	N/A	complex AFL (reABL)
14	65	M	No	Yes	68.2	1414	+	+	0.0405	53.0	150.4	50.7	34.6	11.8	11.2	59.4	46.2	12.3	PVI	N/A	FAT (reABL)
15	68	M	No	Yes	N/A	N/A	+	+	0.1182	50.1	481.1	48.0	32.0	17.0	18.0	51.8	51.3	20.9	PVI	N/A	AT/AFL (PM)
16	70	M	No	Yes	79.0	1457	+	+	0.0688	46.5	637.3	35.0	28.0	13.0	14.0	48.0	46.0	20.0	PVI	N/A	AT (ECG)
																					
17	73	M	No	No	58.0	1449	+	+	0.0200	65.1	149.7	41.0	27.0	13.0	14.0	60.0	36.0	11.2	PVI, AT	FAT	
18	67	M	No	No	N/A	N/A	+	+	0.0407	83.0	195.4	44.1	32.4	13.8	14.4	55.0	53.2	21.1	PVI	N/A	
19	68	M	No	No	58.0	1403	+	+	0.0617	75.0	130.7	43.1	36.5	15.6	16.3	52.1	45.6	12.9	PVI	N/A	
20	74	M	No	No	42.6	1425	+	+	0.0428	45.0	339.9	36.3	27.6	18.9	17.5	56.4	47.7	19.1	PVI	N/A	
21	75	F	No	No	51.0	1436	+	+	0.0459	59.0	170.5	33.2	25.2	13.2	17.5	46.5	43.5	24.1	PVI	N/A	
22	76	M	No	No	N/A	N/A	+	+	0.0234	51.0	206.5	44.7	35.7	14.8	15.0	53.4	47.0	15.5	PVI	N/A	
23	69	M	No	No	56.0	1488	+	+	0.0300	72.0	147.9	47.4	37.3	14.4	15.5	38.1	45.0	12.4	PVI	N/A	
24	78	M	No	No	N/A	N/A	+	N/A	0.0218	89.0	80.9	47.4	34.5	15.0	14.2	43.7	51.5	6.8	PVI	N/A	
25	75	M	No	No	N/A	N/A	+	+	0.0640	47.0	213.8	46.7	40.5	16.7	17.9	37.8	42.7	18.2	PVI, box	N/A	
26	73	F	No	No	55.0	1485	+	+	0.0360	56.0	321.5	39.3	32.0	12.3	11.0	47.6	45.2	10.3	PVI, SVC	−	
27	73	M	No	No	77.0	1524	+	+	0.0227	54.0	126.1	38.6	29.1	16.9	16.9	49.7	35.9	23.3	PVI	−	
28	69	M	No	No	70.1	1448	+	+	0.0420	66.0	53.2	44.2	37.2	17.3	19.5	49.9	36.9	12.3	PVI, non-PV foci	−	
29	74	M	No	No	48.0	1497	+	+	0.0500	55.0	96.7	49.3	39.9	13.4	15.3	40.3	47.1	13.1	PVI	−	

Box, left atrial posterior wall box isolation; PM, pacemaker; roof, left atrial roof line ablation; other abbreviations as in *Tables 1, and 3-5. 1*, *2*, *5*, and *7*.

**Table 7:** Characteristic of ATTRwt-CM patient who underwent catheter ablation for focal AT

Patient no.	Age/sex (years)		Recurrence of FAT	Paroxysmal /persistent	MRI-ECV (%)	Native T_1_ (ms)	PyP	Biopsy	hs-cTnT (ng/mL)	eGFR (mL/min /1.73 m^2^)	BNP (pg/mL)	LVDd (mm)	LVDs (mm)	IVSTd (mm)	PLVWd (mm)	LVEF (%)	LADs (mm)	*E*/*e*′	Ablation lesions	Sustained non-CTI–dependent AFL/FAT remaining or induced at the end of the session	Recurrence arrhythmia
AV annulus focal AT																					
1	67	M	No	Paroxysmal	63.0	1440	+/	+	0.0536	57.0	254.8	44.5	35.5	13.7	15.9	37.9	43.9	10.1	Focal AT (TV10)	−	
Crista terminalis focal AT																					
2	73	M	No	Persistent	58.0	1449	+/	+	0.0200	65.1	149.7	41.0	27.0	13.0	14.0	60.0	36.0	11.2	CT focal AT	−	
3	79	M	No	N/A	N/A	N/A	+/	+	0.1015	68.1	97.1	32.0	20.0	15.0	16.0	64.7	56.1	19.5	CT focal AT	−	AF
4	79	M	No	Persistent	N/A	N/A	+/	+	0.0700	44.0	562.7	51.6	38.1	12.2	12.3	49.8	44.5	11.8	CT focal AT	−	AFL (reABL)
5	68	M	Yes	Paroxysmal	80.0	1504	+/	+	0.0283	82.8	131.2	47.0	31.9	12.5	12.2	58.6	39.8	13.4	CT focal AT	AT	AT (reABL)
	73	M	No	Paroxysmal	80.0	1504	+/	+	0.0280	63.0	91.4	46.5	36.7	12.9	14.3	56.4	41.0	15.8	CT focal AT	−	
																					
Other focal AT (multiple focal ATs)																					
6	83	M	Yes	Persistent	N/A	N/A	+/	+	0.0448	50.0	151.3	44.9	40.2	12.2	11.4	31.8	40.2	9.7	Multiple FATs (CT, AT2 unmap)	– (nonsus)	AT (reABL)
	83	M	Yes	Persistent	N/A	N/A	+/	+	0.1860	51.0	166.0	45.0	41.0	12.1	12.2	34.9	42.8	9.7	Multiple FATs (AT1, AT2, AT3 unmap)	AT	AT/AFL (ECG)
7	72	M	Yes	Paroxysmal	N/A	N/A	+/	+	0.0418	54.6	185.8	40.1	29.8	13.4	13.1	53.6	40.1	18.1	Multiple FATs (CSos, RA ant-sept, CSos2)	−	AT (reABL)
	72	M		Paroxysmal	N/A	N/A	+/	+	0.1010	49.0	185.8	40.9	34.9	13.1	13.5	43.3	42.8	20.6	AVN ablation		AT (PM)
8	78	M	Yes	Persistent	89.0	1444	+/	+	0.0612	56.0	879.9	47.5	41.4	15.2	15.8	41.4	51.7	20.8	Multiple FATs (TV12, TV9:30, TV8, TV10:30)	AT	AT (reABL)
	78	M	Yes	Persistent	89.0	1444	+/	+	0.1215	66.0	496.5	46.2	40.5	14.1	16.1	35.9	43.9	11.1	multiple FATs (CSos, His, LIPV post, TV7, RA late)	AT	AT (reABL)
	78	M		Persistent	89.0	1444	+/	+	0.0964	69.0	672.1	42.5	39.1	13.8	19.3	36.3	54.0	15.0	AVN ablation		AT (PM)
9	78	F	Yes	Persistent	N/A	N/A	+/	+	0.0580	26.5	71.1	41.0	34.0	16.0	16.0	46.0	44.0	31.7	CSos FAT	−	AT/AFL (PM)
10	63	M	Yes	N/A	69.0	1451	+/	+	0.0717	54.0	589.7	41.1	32.8	14.5	15.3	55.0	46.5	18.7	Multiple FATs (LA septum, MV2, LAA)	AT	AT (ECG)
11	87	M	Yes	Persistent	N/A	N/A	+/	+	0.0234	60.0	116.7	45.0	37.9	15.7	16.2	47.3	45.5	6.6	multiple FATs (RA1, RA2, RA3)	AT	AT (reABL)
	87	M		Persistent	N/A	N/A	+/	+	0.0234	60.0	116.7	45.0	37.9	15.7	16.2	47.3	45.5	6.6	AVN ablation		AT/AFL (PM)
12	78	F	Yes	N/A	N/A	N/A	+/	+	0.0132	46.0	266.2	46.1	36.8	10.0	9.5	45.0	43.5	27.6	LA posterior FAT	−	AT/AFL (ECG)
13	71	M	Yes	Persistent	45.5	1300	+/	+	0.0468	37.0	401.7	43.4	31.5	12.4	12.2	54.4	28.4	21.6	LA inf-post FAT	−	AT (ECG)
14	72	M	No	Paroxysmal	64.8	1456	+/	+	0.0578	49.0	122.2	40.3	28.1	21.1	21.1	64.2	43.4	18.5	Multiple FATs (RAA, His, LAA)	−	
15	82	M	No	Paroxysmal	39.3	1347	+/	N/A	0.0374	35.0	89.6	39.5	28.2	15.6	18.5	53.7	42.6	18.3	Multiple FATs (CT, CSos)	−	
16	80	M	Yes	Persistent	69.3	1431	+/	+	0.0644	53.0	77.1	39.3	30.0	13.5	16.3	51.6	40.9	20.1	Multiple FATs (RAA1, RAA2, RAA3, RAA4)	FAT	AT (ECG)
	81	M		Persistent	69.3	1431	+/	+	0.0790	30.0	135.2	38.6	30.0	13.7	16.7	46.6	39.0	13.2	AVN ablation		AT (PM)
17	66	M	No	Paroxysmal	68.2	1414	+/	+	0.0586	51.0	269.6	42.6	33.1	13.2	13.5	47.0	47.8	12.3	Multiple FATs (CSos1, CSos2)	N/A	
18	78	M	Yes	Persistent	65.4	1381	+/	+	0.0686	48.0	582.6	41.6	34.6	13.8	14.4	40.6	40.1	14.5	Multiple FATs (His1, His2)	N/A	AT/AFL (ECG)

ant-sept, anterior septum; AV, atrioventricular; AVN, atrioventricular node; CSos, coronary sinus ostium; CT, crista terminalis; His, His bundle; inf-post, inferoposterior; LAA, left atrial appendage; late, lateral; LIPV post, left inferior pulmonary vein posterior; MV, mitral valve annulus (o’clock); nonsus, non-sustained; TV, tricuspid valve annulus (o’clock); unmap, unmappable; other abbreviations as in *Tables 1, and 3–6.*

**Table 8:** Univariate and multivariate logistic regression analysis for occurrence of complex AFL or multiple focal AT in patients with ATTRwt-CM

	Univariate logistic regression			Multivariate logistic regression		
Variable	OR	95% CI	*P* value	OR	95% CI	*P* value
Age (per year)	0.988	0.901–1.083	0.792			
Gender, male (yes)	1.000	0.165–6.052	1.000			
Height (per cm)	1.066	0.978–1.162	0.149			
Weight (per kg)	0.941	0.881–1.006	0.074			
MRI-ECV (per %)	1.091	1.019–1.168	0.012	1.077	1.001–1.159	0.047
Native T_1_ (per ms)	1.002	0.989–1.015	0.778			
hs-cTnT (per ng/dL)	1.286	0.990–1.671	0.060			
eGFR (per mL/min/1.73 m^2^)	0.981	0.940–1.025	0.401			
BNP (per pg/mL)	1.004	1.001–1.007	0.021	1.006	0.999–1.013	0.086
LVDd (per mm)	1.067	0.956–1.191	0.247			
LVDs (per mm)	1.109	0.994–1.237	0.064			
IVSTd (per mm)	0.847	0.654–1.098	0.210			
PLVWd (per mm)	0.845	0.670–1.066	0.155			
LVEF (per %)	0.963	0.906–1.024	0.231			
LADs (per mm)	0.944	0.858–1.038	0.233			
*E*/*e*′ ratio (per)	0.964	0.888–1.047	0.387			

OR, odds ratio; other abbreviations as in *Table 1–3*. Supplementary material online, *Tables S1* and *S2*.

Catheter ablation was performed using EnSite system (Abbott Laboratories, Abbott Park, IL) or CARTO system (Biosense Webster, Irvine, CA) as 3D mapping systems, with multipolar catheters such as Advisor HD Grid catheter (Abbott Laboratories) and PENTARAY catheter (Biosense Webster), respectively. During the period of this study from May 2017 to June 2022, all patients underwent CA using TactiCath SE (Abbott Laboratories) or THERMOCOOL SMARTTOUCH SF (Biosense Webster) with irrigated contact force sensing as ablation catheter, and energy deliver was performed at 25–35 W in all cases, with the index standardized to lesion size index ≥ 5.2^19^ or ablation index ≥ 400^20^ for ablation of atrial muscle, but not high power short duration.

Catheter ablation for AF was performed with extensive encircling of ipsilateral pulmonary vein isolation with a 4 mm tag partially overlapped with reference to the CLOSE protocol.^20^ After that, isoproterenol infusion (4 µg/min) and adenosine 20 mg bolus were performed to induce non-pulmonary vein (PV) triggers.^13^ If the non-PV triggers originated from the superior vena cava (SVC), SVC isolation was performed, and if it originated from the left atrial posterior wall, box isolation was performed. If non-PV triggers were induced from within the atrium, CA to the same site was performed. Catheter ablation of AF, whether paroxysmal or persistent, is standardized in the same way with trigger exclusion in all patients, and additional ablation for substrates is not routinely performed. Atrial flutter ablation was also performed using a 3D mapping system to identify the circuits and create lines to intercept the macroreentry circuits. For example, linear ablation of the CTI was performed in the CTI-dependent AFL; linear ablation of the mitral isthmus (left inferior PV to the mitral valve) was performed in the mitral AFL; and linear ablation across the SCZ was performed in the AFL via the SCZ in the low-voltage area in the atrium. Atrial tachycardia was also identified as the earliest atrial activation site (EAAS) using a 3D mapping system, and ablation of the EAAS and surrounding area was performed. In the ablation of the AF and AFL, if sustained AT or a different AFL appeared or was induced during the procedure, treatments for sustained AT or AFL were also performed. The endpoint of ablation for AT and AFL was to terminate tachycardia and/or prevent sustained AT/AFL as much as possible.

The follow-up evaluation methodology was consistent, and complete follow-up was performed without loss of cases. All patients, whether CA was performed or not, were followed up at an outpatient amyloidosis clinic once a year after the diagnosis of ATTRwt-CM, and 12-lead ECG was always performed at that time. Furthermore, all patients who underwent CA were visited in the outpatient clinic at 1, 3, 6, and 12 months after the CA procedure for a detailed post-operative assessment of arrhythmia recurrence. Specifically, recurrence was assessed using 12-lead ECG (at 1, 3, 6, and 12 months), Holter ECG (or 1-week Holter ECG from 2020 onward, at 6 and 12 months), and records of pacemakers or other devices if present (at 1, 3, 6, and 12 months). Post-operative antiarrhythmic drugs, including β-blockers, were not administered because of amyloidosis. Subsequently, all patients continued to visit our clinic at least once or twice a year again for follow-up of ATTRwt-CM and arrhythmias, with 12-lead ECG evaluation at each visit. If the arrhythmia recurred or HF developed, the patient made an unscheduled visit to the clinic for appropriate evaluation and treatment.

### Study outcomes

The outcomes of this study were all-cause mortality, cardiovascular mortality, and HF hospitalization from disease diagnosis between ATTRwt-CM patients with and without CA for AF/AFL/AT, the presence or absence of arrhythmia recurrence after CA for AF/AFL/AT, details of the recurrent arrhythmia, clinical course of recurrence for each arrhythmia, and factors associated with recurrence to establish the optimal therapy for ATTRwt-CM with AF/AFL/AT, especially stratifying patients for whom CA should be recommended. Because some patients had multiple arrhythmias in a single patient and the timing of CA for each was different, to make the real-world clinical practice easier to understand, the outcomes of CA for AF/AFL/AT were also investigated separately for each arrhythmia, with recurrence of that arrhythmia from the time of each CA. These outcomes were shown with a 5-year follow-up period, as described in the Supplementary material.

### Statistical analysis

Details of the statistics are noted in the Supplementary material.

## Results

### Patient background

Patient characteristics are shown in *Table 1*. There were significant differences in age, New York Heart Association (NYHA), comorbid arrhythmia, systolic blood pressure, high-sensitivity cardiac troponin T (hs-cTnT), BNP levels, estimated glomerular filtration ratio (eGFR), left atrial end-systolic diameter, deceleration time (Dct), and the use of mineralocorticoid receptor antagonists, β-blockers, and loop or thiazide diuretics between patients without AF/AFL/AT, patients with AF/AFL/AT but without ablation, and patients with AF/AFL/AT but with ablation. There were internal correlations between NYHA and age, hs-cTnT, BNP levels, and eGFR [correlation coefficient was 0.430 (*P* < 0.001), 0.439 (*P* < 0.001), 0.476 (*P* < 0.001), and −0.348 (*P* < 0.001), respectively] and between loop or thiazide diuretics and mineralocorticoid receptor antagonist [correlation coefficient was 0.301 (*P* < 0.001); *Table 2*].

### Prognosis and outcomes of catheter ablation cases for atrial fibrillation/atrial flutter/atrial tachycardia in wild-type transthyretin amyloid cardiomyopathy

**Figure 3:**
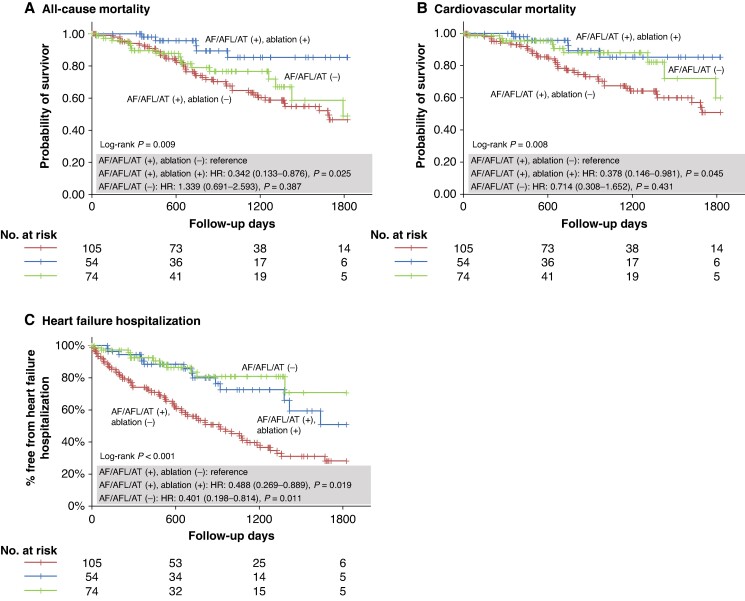
Study outcomes regarding patient prognosis. The comparison of all-cause mortality (*A*), cardiovascular mortality (*B*), and heart failure hospitalization (*C*) between ATTRwt-CM patients with AF/AFL/AT who underwent catheter ablation (blue), ATTRwt-CM patients with AF/AFL/AT who did not underwent catheter ablation (red), and ATTRwt-CM patients without AF/AFL/AT (green) was shown. AF, atrial fibrillation; AFL, atrial flutter; AT, atrial tachycardia; ATTRwt-CM, wild-type transthyretin amyloid cardiomyopathy; HR, hazard ratio.

**Figure 4:**
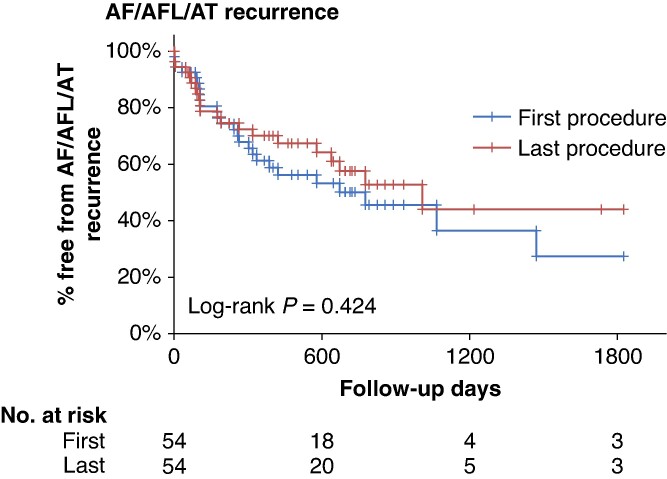
Study outcomes regarding arrhythmia recurrence after catheter ablation. The recurrence-free rate of AF/AFL/AT overall in wild-type transthyretin amyloid cardiomyopathy patients after first procedure of catheter ablation and those after last procedure of catheter ablation was shown. AF, atrial fibrillation; AFL, atrial flutter; AT, atrial tachycardia.

After being adjusted for non-internal correlated factors among these significantly different parameters (NYHA, Dct, β-blockers, and loop or thiazide diuretics) to eliminate differences in patient background, multiple Cox regression analysis and Kaplan–Meier analysis revealed that, in patients with concomitant AF/AFL/AT, CA significantly improved all-cause mortality [hazard ratio (HR): 0.342, 95% confidence interval (CI): 0.133–0.876, *P* = 0.025], cardiovascular mortality (HR: 0.378, 95% CI: 0.146–0.981, *P* = 0.045), and HF hospitalization (HR: 0.488, 95% CI: 0.269–0.889, *P* = 0.019) compared with those without CA, and especially HF hospitalization was improved to the same level as patients without AF/AFL/AT (*Figure 3* and *Table 2*). The recurrence-free rate of AF/AFL/AT overall in ATTRwt-CM patients after the first session of CA was 61.3% at 1-year, 50.2% at 2-year, and 27.4% at 5-year follow-up, but that after final CA was 70.1% at 1-year, 57.6% at 2-year, and 44.0% at 5-year follow-up (*Figure 4*). There were no complications such as cardiac tamponade, symptomatic stroke/transient ischaemic attack, pericarditis, or atrioesophageal fistula.

### Cavotricuspid isthmus–dependent atrial flutter

**Figure 5:**
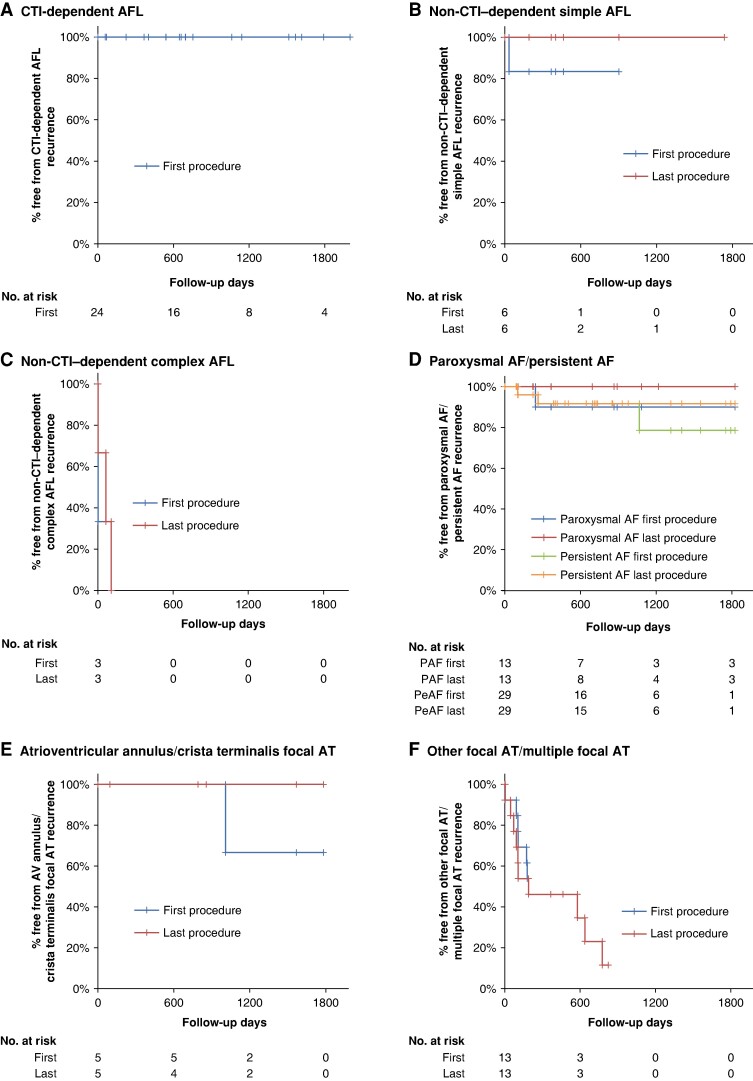
Incidence of free from recurrence after catheter ablation in each arrhythmia. The incidence of free from recurrence after catheter ablation in each arrhythmia in patients with wild-type transthyretin amyloid cardiomyopathy is shown in (*A*) CTI-dependent AFL, (*B*) non-CTI–dependent simple AFL, (*C*) non-CTI–dependent complex AFL, (*D*) paroxysmal or persistent AF, (*E*) focal AT originating from AV annulus, or crista terminalis, and (*F*) focal AT originating from other sites or multiple focal ATs. The line of first procedure is the result after the initial session, and the line of last procedure is the final result after multiple sessions in each arrhythmia. AF, atrial fibrillation; AFL, atrial flutter; AT, atrial tachycardia; AV, atrioventricular; CTI, cavotricuspid isthmus; PAF, paroxysmal AF; PeAF, persistent AF.

Among the 54 ablation patients, 24 underwent linear ablation of the CTI for CTI-dependent AFL (*Figure 2*); a representative case of CTI-dependent AFL is shown in Supplementary material online, *Figure S1*. Of the 24 patients (*Table 3*), recurrence of CTI-dependent AFL did not occur in any patients, regardless of whether it was paroxysmal or persistent (*Figure 5A*). In addition, CTI reconnection was evaluated in six of the eight patients who underwent a second session due to other arrhythmias, but all six of the patients did not have CTI reconnection (patient nos. 2, 7, 13, 15, 17, and 18 in *Table 3*). In contrast, 13 of the 24 patients had a recurrence of AF, non-CTI–dependent AFL, or AT after ablation of CTI-dependent AFL, and all five patients with sustained non-CTI–dependent AFL or focal AT remaining or induced at the end of the session had a recurrence of these arrhythmias (patient nos. 1–5 in *Table 3*). Eventually, except for patient no. 1 in *Table 3*, whose AFL was mitral (simple AFL), and patient no. 2 in *Table 3*, whose focal AT originated from the crista terminalis, AFL/AT recurred repeatedly. Conversely, all patients who did not induce sustained non-CTI–dependent AFL or sustained focal AT at the end of CTI-dependent AFL ablation did not subsequently develop these arrhythmias (patient nos. 6–14 in *Table 3*), but three patients subsequently developed AF (patient nos. 6–8 in *Table 3*). In addition, 10 patients did not undergo tachycardia induction at the end of CTI-dependent AFL ablation (patient nos. 15–24 in *Table 3*), but 5 of them had AF/non-CTI–dependent AFL/focal AT after ablation (patient nos. 15–19 in *Table 3*). Some of these patients probably would have induced these arrhythmias once they underwent tachycardia induction, but in any case, there are no recurrences of CTI-dependent AFL in all 24 cases (*Table 3*).

### Non-cavotricuspid isthmus–dependent atrial flutter

Nine of the 54 patients underwent CA for non-CTI–dependent macroreentrant tachycardia (AFL; *Figure 2* and *Table 4*). Non-CTI–dependent simple AFL, such as mitral AFL and left atrial anterior channel–dependent AFL, was found in six of the nine cases (patient nos. 1–6 in *Table 4*), and AFL recurrence due to reconnection was observed in one case (patient no. 1 in *Table 4*). However, AFL recurrence was not observed after reablation, and there was no recurrence of non-CTI–dependent simple AFL after multiple sessions (*Figure 5B*). A representative case of non-CTI–dependent simple AFL is shown in Supplementary material online, *Figure S2*.

On the other hand, complicated non-CTI–dependent macroreentrant AFL (complex AFL) was observed in three of the nine patients (patient nos. 7–9 in *Table 4*); however, all of them had recurrence with another AFL or arrhythmia thought to be AFL even after multiple ablation sessions (*Figure 5C*). In particular, one patient (patient no. 8 in *Table 4*) had a recurrence of AFL even though it was no longer inducible at the end of the ablation. Although it was not inducible again at the time of reablation, further different arrhythmia thought to be AFL still occurred after multiple sessions. A representative case of non-CTI–dependent complex AFL is presented in Supplementary material online, *Figure S3*.

For some reason, focal AT was rarely seen in non-CTI–dependent AFL, with only one case of focal AT originating from the coronary sinus ostium (CSos; patient no. 4 in *Table 4*).

### Paroxysmal atrial fibrillation

Of the 54 patients, 13 underwent CA for paroxysmal AF (*Figure 2* and *Table 5*). Atrial fibrillation was resolved after ablation, and 11 of the 13 patients had no recurrence of AF after the first session, although one patient (patient no. 2 in *Table 5*) underwent a second ablation for right superior PV reconnection and subsequently had no recurrence of AF, and another patient (patient no. 1 in *Table 5*) had no symptoms of recurrent AF and no desire for ablation. Therefore, except for 1 patient who did not receive the second ablation session, all 12 of the 13 patients with paroxysmal AF were free of AF itself after multiple sessions (*Figure 5D*). A representative case of paroxysmal AF is presented in Supplementary material online, *Figure S4*.

In contrast, sustained focal AT occurred in four patients (patient nos. 3–6 in *Table 5*) during the ablation procedure, and in all cases, AT or arrhythmia thought to be AT recurred after the procedure despite additional ablation for AT.

### Persistent atrial fibrillation

Among the 54 patients, 29 underwent ablation for persistent AF (*Figure 2* and *Table 6*). Atrial fibrillation itself was also resolved after ablation, and only 3 of the 29 patients had AF recurrence (patient nos. 1–3 in *Table 6*), but 1 of them underwent a second session with left atrial posterior wall box isolation because there was no reconnection of PV, with no recurrence thereafter (*Figure 5D*). A representative case of persistent AF is presented in Supplementary material online, *Figure S5*.

However, many patients (13 of 29 patients; patient nos. 4–16 in *Table 6*) had a recurrence of AT/AFL instead of AF after the first ablation session. Because four of them had PV reconnection (patient nos. 4, 5, 12, and 14 in *Table 6*) and two of them had left atrial posterior wall box reconnection (patient nos. 5 and 12 in *Table 6*), all these patients underwent reisolation to eliminate triggers. Nevertheless, if multiple focal ATs or complex AFL occurred during the ablation procedure (10 patients: patient nos. 6–14 and 17 in *Table 6*) and even if ablation for it was performed, AT/AFL recurred in many cases (8 of the 10 patients: patient nos. 6–13 in *Table 6*) except for two focal ATs (patient no. 14: CSos origin focal AT and no. 17: crista terminalis origin focal AT). On the other hand, two patients with simple AFL [patient no. 4: mitral AFL and left atrium anterior channel–dependent AFL and no. 5: mitral AFL (twice due to reconnection)] underwent ablation for AFL, and subsequently, their AFL did not recur.

Eventually, 16 of the 29 patients had AF/AFL/AT recurrence after the first ablation session, while the remaining 13 had no recurrence after the initial treatment (patient nos. 17–29 in *Table 6*). Of these 13 patients, only 1 showed AT originating from the crista terminalis during the ablation procedure (patient no. 17 in *Table 6*). Univariate and multivariate Cox regression analyses showed that higher hs-cTnT levels and the induction of sustained non-CTI–dependent AFL/focal AT were significant parameters for AF/AFL/AT recurrence in patients with persistent AF and ATTRwt-CM (see Supplementary material online, *Table S1*).

### Focal atrial tachycardia

**Figure 6:**
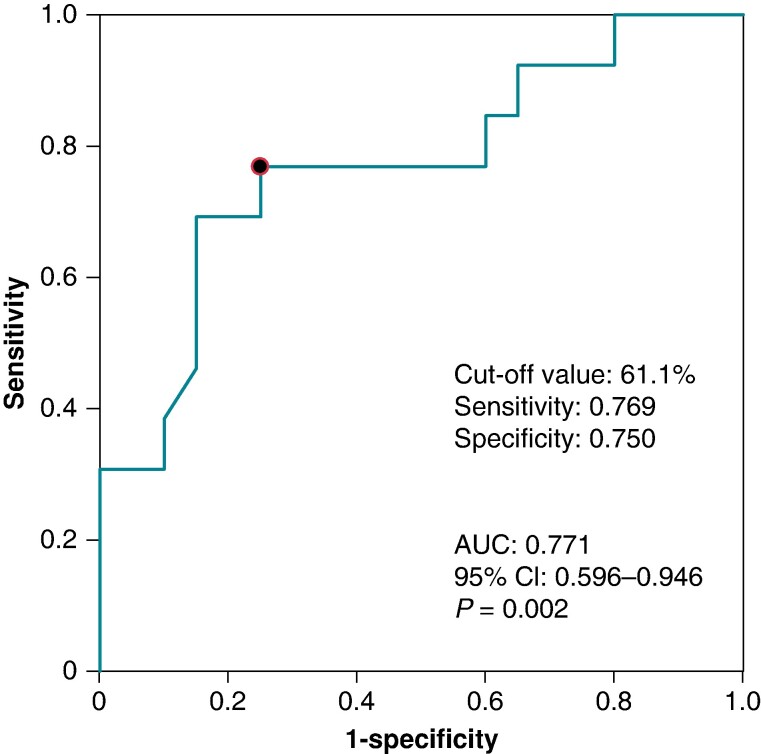
Receiver operating characteristic curve analysis of MRI-ECV. Receiver operating characteristic curve analyses of MRI-ECV in patients with wild-type transthyretin amyloid cardiomyopathy are shown. AUC, area under the curve; CI, confidence interval; MRI-ECV, extracellular volume fraction in magnetic resonance imaging.

Eighteen of the 54 patients underwent ablation for focal AT (*Figure 2* and *Table 7*). Atrioventricular annulus focal AT (patient no. 1 in *Table 7*) and crista terminalis focal AT (patient nos. 2–5 in *Table 7*) did not recur after CA, whether paroxysmal or persistent, while AT originating from the crista terminalis recurred in one case but did not recur with subsequent treatment (*Figure 5E*). In contrast, other focal ATs, such as CSos origin AT and multiple focal ATs, recurred in most cases even if sustained ATs were no longer induced at the end of the ablation session (*Figure 5F*). Multiple focal AT and complex non-CTI–dependent AFL are independently associated with higher extracellular volume fraction in magnetic resonance imaging (cut-off value: 61.1%, sensitivity: 0.769, specificity: 0.750, and area under the curve: 0.771, *P* = 0.002, *Table 7* and *Figure 6*) and might be more likely to occur in advanced ATTRwt-CM. However, while all patients with persistent AT had a recurrence, two of the three patients with paroxysmal AT did not have a recurrence. Univariate and multivariate analyses showed that persistent form of AT and multiple focal ATs were the most significant factors associated with AT or arrhythmia thought to be AT recurrence in patients with ATTRwt-CM (see Supplementary material online, *Table S2*). A representative case of troublesome multiple focal ATs, a characteristic finding of ATTRwt-CM, is shown in Supplementary material online, *Figure S6*.

## Discussion

This study reports the results of CA for AF/AFL/AT in patients with ATTRwt-CM. The recurrence-free rate after CA for atrial arrhythmias complicated with ATTRwt-CM was 70.1% at 1 year, 57.6% at 2 years, and 44.0% at 5 years, even after multiple sessions, but CA significantly reduced all-cause mortality, cardiovascular mortality, and HF hospitalization compared with patients with AF/AFL/AT who did not undergo CA, without complications. For each arrhythmia, extremely poor ablation outcomes were found in complex non-CTI–dependent AFL or multiple focal ATs, but not in other AF/AFL/AT cases.

It has been reported that AF does not affect prognosis in cardiac amyloidosis,^8,21,22^ and in fact, the results of the present study showed no significant difference in prognosis between patients with AF/AFL/AT who did not undergo CA and those without AF/AFL/AT. However, HF hospitalization in patients without AF/AFL/AT was significantly lower than those with AF/AFL/AT but without CA (HR: 0.401, 95% CI: 0.198–0.814, *P* = 0.011, *Figure 3C*), and consequently, cardiovascular mortality tended to be lower (HR: 0.714, 95% CI: 0.308–1.652, *P* = 0.431, *Figure 3B*). Both the group without AF/AFL/AT and the group with AF/AFL/AT but without CA were significantly equally older than the group with ablation for AF/AFL/AT, which may be one of the reason why there was no significant difference in all-cause mortality, probably including non-cardiac death (HR: 1.339, 95% CI: 0.691–2.593, *P* = 0.387, *Figure 3A*) and cardiovascular mortality (HR: 0.714, *P* = 0.431, *Figure 3B*) between the group without AF/AFL/AT and the group with AF/AFL/AT but without CA, and why the group with ablation had the best prognosis over the group without AF/AFL/AT. Furthermore, patients with AF/AFL/AT but without CA included those who were asymptomatic with no HF exacerbations and had chronic arrhythmia at the time of diagnosis, suggesting that AF/AFL/AT may not have an impact on the prognosis of these asymptomatic chronic patients. However, all-cause mortality, cardiovascular mortality, and HF hospitalization were most favourable with CA in patients with AF/AFL/AT who were symptomatic or had HF exacerbations, suggesting for the first time, to our knowledge, that prognosis and HF hospitalization in ATTRwt-CM patients with symptomatic or HF exacerbations of AF/AFL/AT may be improved with feasible and safe CA.

On the other hand, there are a few small case reports detailing CA for AF/AFL/AT complicated by cardiac amyloidosis.^23–26^ Most of them did not have good outcomes, and Barbhaiya *et al*.^25^ reported that atrial voltage in amyloidosis patients was lower than that in non-amyloidosis patients and AT was induced more frequently. However, Donnellan *et al*.^24^ showed the usefulness of CA in patients without evidence of advanced amyloidosis, and Tan *et al*.^26^ also reported that AV node ablation improved NYHA to a similar level compared with the ablation group (for more details, please refer to the Supplementary material).

**Figure 7:**
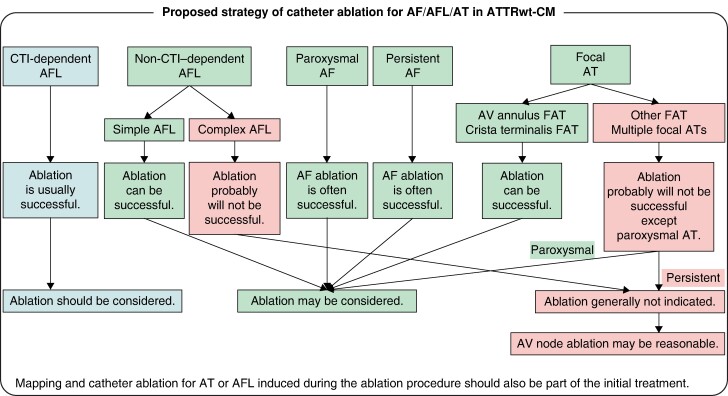
Proposed strategy of catheter ablation for AF/AFL/AT in ATTRwt-CM. Proposed strategy of catheter ablation for AF/AFL/AT in ATTRwt-CM was shown. AF, atrial fibrillation; AFL, atrial flutter; AT, atrial tachycardia; ATTRwt-CM, wild-type transthyretin amyloid cardiomyopathy; AV, atrioventricular; CTI, cavotricuspid isthmus; FAT, focal atrial tachycardia.

Hence, in this article, we present a proposed strategy of CA for AF/AFL/AT in ATTRwt-CM patients to reduce HF hospitalizations and improve prognosis, despite a conditional view based on a limited number of cases (*Figure 7*). (i) First of all, ablation should be considered for CTI-dependent AFL because ablation is usually successful. Furthermore, ablation may be considered for a non-CTI–dependent AFL if it is a simple AFL because ablation can be successful. However, ablation is unsuccessful in complex AFL, even in non-CTI–dependent AFL. Although a simple macroreentrant AFL can be terminated with a single line creation, a complex AFL that cannot be terminated with one linear ablation may recur even after the AFL is no longer induced and may be difficult to control. This may be a unique characteristic of ATTRwt-CM; therefore, ablation is generally not indicated for complex AFL, and AV node ablation may be reasonable if necessary. In addition, CTI-dependent AFL is not usually accompanied by CTI conduction recurrence itself; however, the co-occurrence of persistent focal AT (except for AV annulus AT and crista terminalis AT) and complex AFL during the ablation procedure can be troublesome, even for CTI-dependent AFL. (ii) Second, ablation of the AF, whether paroxysmal or persistent, may be considered since ablation of the AF itself is often successful. AF itself can be controlled by CA; however, its recurrence is dependent on AT or AFL. Atrial fibrillation recurrence, including AFL and AT, was more likely when focal AT or non-CTI–dependent AFL was induced during the ablation procedure (see Supplementary material online, *Table S1*). Although it is still worth ablation for AV annulus focal AT, crista terminalis focal AT, or simple AFL, it is difficult to perform for other ATs including multiple focal ATs or complex AFL, which tend to occur in advanced cases and often recurs with AFL or AT. (iii) Finally, ablation of focal ATs may be considered if they are AV annular focal AT or crista terminalis focal AT, as ablation can be successful, but if they are other focal ATs or multiple focal ATs, especially if they are persistent, ablation will probably not be successful (see Supplementary material online, *Table S2*). Therefore, ablation is generally not indicated, and AV node ablation may be reasonable if necessary. However, while all patients with persistent other ATs had a recurrence, two of the three patients with paroxysmal other ATs did not have a recurrence, suggesting that it might be possible to control paroxysmal other focal ATs. Therefore, even for other ATs, including multiple focal ATs, if it is paroxysmal, ablation may be considered, because it can be successful (*Figure 7*).

It is important to note that it is not necessary to be pessimistic about ablation for ATTRwt-CM, but the success rate of ablation for persistent complex AFL or multiple focal ATs is extremely low. Thus, once they are encountered, a graceful change in strategy to AV node ablation without excessive attempts may be a better approach. However, AT or AFL induced during the ablation procedure will undoubtedly occur later. Since simple AFL and some AT can be cured completely, mapping and ablation for induced AT or AFL should be performed as part of the initial treatment (*Figure 7*).

In addition, it has been reported that 35% of patients diagnosed with ATTRwt-CM previously had a diagnosis with hypertrophic cardiomyopathy, hypertensive heart disease, or some other diagnosis except cardiac amyloidosis,^4^ and it is not rare to take 1–2 years from the onset of symptoms to diagnosis, receive treatment based on a false diagnosis, or wait too long before diagnosis without suspecting ATTRwt-CM.^3^ Because AF/AFL/AT associated with ATTRwt-CM is characterized as an arrhythmia, some of which are intractable and some of which are controllable, it is extremely important to diagnose ATTRwt-CM in advance and to recognize whether AF/AFL/AT is associated with ATTRwt-CM.

However, the arrhythmia mechanism was not fully investigated in this study. In other words, the deposition of TTR amyloid in the atrial muscle of ATTRwt-CM has not been investigated pathologically or histologically, and it is not clear how the arrhythmia circuit is organized in the atrial muscle, which is known to have a multilayered structure,^27,28^ or why the ablation success rate is poor in some arrhythmias. Similarly, atrial voltage maps were not available for all patients, resulting in missing data; therefore, electrophysiological analysis of the mechanisms of arrhythmias could not be performed. Further studies are required, especially regarding the electrophysiological evaluation using this voltage map, which is the next issue to be addressed.

It has been reported that patients who took tafamidis after ablation for AF had a higher post-operative sinus rhythm maintenance rate than those who did not take tafamidis,^29^ but in this study, the effect of tafamidis was not examined because the timing of the start of tafamidis administration varied from case to case and the significance of the results differed depending on the type of arrhythmia. Thirty-eight of the 54 patients (70%) who underwent CA for AF/AFL/AT ultimately took tafamidis, but there was no significant difference in the rate of maintenance of sinus rhythm with or without tafamidis [22/38 (58%) vs. 10/16 (63%), *P* = 0.753]. Further investigation is necessary because the inhibition of ATTRwt-CM progression^30^ may be extremely important in the pathophysiology of arrhythmias and the prevention of AF/AFL/AT recurrence.

### Limitation

This is a single-centre cohort study and was not randomized. The decision to perform CA for AF/AFL/AT was made in accordance with the guidelines, primarily for symptomatic or HF-causing cases, but the treatment strategy also took into account patient intent and physician discretion, and there was a bias in the grouping of patients who underwent CA or not. Some patients did not wish to undergo CA despite indications, and it is possible that elderly patients with end-stage HF were not selected for CA. However, HRs were calculated after adjusting for differences in patient background, and the present study is the real-world clinical cohort of CA in ATTRwt-CM. Because ablation and mapping techniques evolve over time, the long duration of this study is a limitation in capturing treatment outcomes. However, the version of the mapping system is updated from time to time, but the multielectrode catheter such as Advisor HD Grid catheter and PENTARAY catheter was already available since 2017, and the accuracy of the mapping system is not so different from today’s system. Some cases underwent CA prior to May 2017, but with current ablation and mapping techniques, the results of CA for ATTRwt-CM could be a little better than the results of this study, and even older mapping and ablation techniques could yield reasonable results with multiple sessions of CA. The results of this study are considered acceptable even today, despite the length duration of the study. However, while annual follow-up is complete including with regard to mortality and HF hospitalization and the evaluation method for arrhythmia recurrence was consistent, there were limitations in detecting paroxysmal asymptomatic arrhythmias on 12-lead ECG or Holter ECG. To detect these arrhythmias as much as possible, a 1-week Holter ECG was used. This study did not quantitatively investigate the improvement in quality of life with CA for AF/AFL/AT. Although the data in this study are insufficient because of the small number of patients, we believe that the study of 54 ATTRwt-CM patients who underwent CA is the largest to date to our knowledge and is fully valuable because of the rarity of this disease. However, it is still conditional due to the small number of cases, and further validation in a larger number of cases is needed. Furthermore, if all patients could be followed for 5 years, it cannot be denied that the results could be different.

## Conclusions

Persistent complex AFL that requires multiple line creations to terminate and multiple focal ATs originating from sites other than the AV annulus and crista terminalis are characteristic arrhythmias found in ATTRwt-CM and have extremely poor treatment outcomes. Catheter ablation for these arrhythmias is almost always ineffective, and AV node ablation is preferred if necessary. In contrast, CA is effective for other AF/AFL/AT and is a useful strategy for patients with ATTRwt-CM who cannot be treated with arrhythmia medications, resulting in improved all-cause mortality, cardiovascular mortality, and HF hospitalization.

## Supplementary Material

euae155_Supplementary_Data

## Data Availability

The data underlying this article cannot be shared publicly due to the privacy of individuals that participated in the study. The data will be shared on reasonable request to the corresponding author.
