# Supplementary material for: Clinical outcomes of catheter ablation for atrial fibrillation, atrial flutter, and atrial tachycardia in wild-type transthyretin amyloid cardiomyopathy: a proposed treatment strategy for catheter ablation in each arrhythmia
Source: Europace. 2024 Jun 27;26(6):euae155. doi: 10.1093/europace/euae155 (PMC11208780; doi:10.1093/europace/euae155)
Supplement: euae155_Supplementary_Data [file euae155_supplementary_data.zip › Supplemental Figure Legends.docx]

**Supplemental Figure Legends**

**Supplemental Figure S1. Representative case of CTI-dependent AFL.**

Panel A is an isochronal map of a case of cavotricuspid isthmus (CTI)-dependent atrial flutter (AFL) in a 64-year-old wild-type transthyretin amyloid cardiomyopathy male (Patient No. 10 in Table 3); AFL was rotated clockwise around the tricuspid annulus and terminated with linear ablation of the CTI (panel B), following no recurrence.

**Supplemental Figure S2. Representative case of non-CTI-dependent simple AFL.**

Panel A is an isochronal map of a case of non-cavotricuspid isthmus (CTI)-dependent simple atrial flutter (AFL) in an 81-year-old wild-type transthyretin amyloid cardiomyopathy male (Patient No. 2 in Table 4) who had previously undergone pulmonary vein isolation, roof line ablation, and complex fractionated atrial electrogram ablation for persistent atrial fibrillation. AFL was rotated counterclockwise around the mitral annulus, with the possibility of bi-atrial tachycardia, but ultimately terminated by mitral isthmus block (from left inferior pulmonary vein to mitral valve) (panel B), following no recurrence.

**Supplemental Figure S3. Representative case of non-CTI-dependent complex AFL.**

Panel A is an isochronal map of a case of non-cavotricuspid isthmus (CTI)-dependent complex atrial flutter (AFL) in an 84-year-old wild-type transthyretin amyloid cardiomyopathy male (Patient No. 7 in Table 4) who had previously undergone pulmonary vein isolation, roof line ablation, and complex fractionated atrial electrogram ablation for persistent atrial fibrillation. AFL appeared to rotate around the scar area at the posterior inferior portion of the left atrium, but did not terminate with multiple lines connecting from the scar area to the posterior wall of the left atrium or mitral annulus (panel B). There were broad low-voltage areas spreading even in the unburned region of the left atrium (panel C), and AFL recurred only two days after catheter ablation.

**Supplemental Figure S4. Representative case of paroxysmal AF.**

Panel A shows the pulmonary vein isolation of a case of paroxysmal atrial fibrillation (AF) in a 59-year-old wild-type transthyretin amyloid cardiomyopathy male (Patient No. 7 in Table 5). As demonstrated in the voltage maps of the left atrium (Panel B) and right atrium (Panel C) of this patient after pulmonary vein isolation, there are some patients with wild-type transthyretin amyloid cardiomyopathy who did not have any low-voltage areas yet, and neither atrial flutter nor atrial tachycardia was induced in this patient, nor recurrence of AF was observed.

**Supplemental Figure S5. Representative case of persistent AF.**

Panel A shows a case of persistent atrial fibrillation (AF) in a 73-year-old wild-type transthyretin amyloid cardiomyopathy female (Patient No. 26 in Table 6). As demonstrated in the voltage maps of the left atrium (Panel A) of this patient after pulmonary vein isolation, even in patients with wild-type transthyretin amyloid cardiomyopathy, persistent AF patients may have more low-voltage areas than paroxysmal AF patients, but some patients, such as this patient, do not have large low-voltage areas, and neither atrial flutter nor atrial tachycardia was induced, nor recurrence of AF was observed. In this patient, triggers derived from the superior vena cava also occurred, therefore, we performed superior vena cava isolation as shown in Panel B (isochronal map during sinus rhythm).

**Supplemental Figure S6. Representative case of multiple focal ATs.**

A case of persistent multiple focal atrial tachycardias (ATs) in an 80-year-old wild-type transthyretin amyloid cardiomyopathy male (Patient No. 16 in Table 7) was shown. Ablation was performed for a focal AT from the right atrial appendage slightly atrial side of the 11 o'clock position of the tricuspid annulus, but the AT did not terminate (panel A). After remapping, the origin of AT had moved toward the septum; therefore, ablation was applied to the area, but the AT still did not terminate (panel B). Hence, another remapping was performed, and the origin was found to have moved deeper into the right atrial appendage this time. Ablation was performed on the earliest activation site; however, the AT did not terminate even then (panel C). Fourth mapping revealed that the origin had migrated to another location in the right atrial appendage, but ablation did not terminate AT eventually (panel D). In multiple focal ATs, no matter how many times ablation is performed, the ATs keep moving and do not terminate, or are induced one after the other, with no end in sight.
