# Supplementary material for: Clinical outcomes of catheter ablation for atrial fibrillation, atrial flutter, and atrial tachycardia in wild-type transthyretin amyloid cardiomyopathy: a proposed treatment strategy for catheter ablation in each arrhythmia
Source: Europace. 2024 Jun 27;26(6):euae155. doi: 10.1093/europace/euae155 (PMC11208780; doi:10.1093/europace/euae155)
Supplement: euae155_Supplementary_Data [file euae155_supplementary_data.zip › Supplemental Table S1.docx]

**Supplemental Table S1. Univariate and multivariate cox regression analysis for recurrence of AF/AFL/AT in persistent AF patients with ATTRwt-CM**

|  | Univariate Cox Regression | | |  | Multivariate Cox Regression | | |  | Multivariate Cox Regression | | |
| --- | --- | --- | --- | --- | --- | --- | --- | --- | --- | --- | --- |
| Variable | HR | 95% Cl | *p* value |  | HR | 95% Cl | *p* value |  | HR | 95% Cl | *p* value |
| Age (per years) | 1.100 | 0.964 – 1.254 | 0.155 |  |  |  |  |  |  |  |  |
| Gender, Male (yes) | 1.597 | 0.358 – 7.118 | 0.539 |  |  |  |  |  |  |  |  |
| MRI-ECV (per %) | 1.059 | 1.004 – 1.116 | 0.034 |  |  | – |  |  |  | – |  |
| Native T1 (per msec) | 0.988 | 0.971 – 1.006 | 0.188 |  |  |  |  |  |  |  |  |
| hs-cTnT (per ng/dL) | 1.017 | 1.005 – 1.028 | 0.009 |  | 1.022 | 1.004 – 1.040 | 0.018 |  |  | – |  |
| eGFR (per ml/min/1.73m^2^) | 0.962 | 0.918 – 1.009 | 0.113 |  |  |  |  |  |  |  |  |
| BNP (per pg/mL) | 1.003 | 1.000 – 1.006 | 0.020 |  |  | – |  |  | 1.003 | 0.999 – 1.006 | 0.163 |
| LVDd (per mm) | 1.000 | 0.899 – 1.112 | 0.995 |  |  |  |  |  |  |  |  |
| LVDs (per mm) | 0.982 | 0.878 – 1.098 | 0.750 |  |  |  |  |  |  |  |  |
| IVSTd (per mm) | 0.882 | 0.683 – 1.140 | 0.338 |  |  |  |  |  |  |  |  |
| PLVWd (per mm) | 0.883 | 0.724 – 1.077 | 0.219 |  |  |  |  |  |  |  |  |
| LVEF (per %) | 1.014 | 0.946 – 1.088 | 0.689 |  |  |  |  |  |  |  |  |
| LADs (per mm) | 1.000 | 0.900 – 1.110 | 0.993 |  |  |  |  |  |  |  |  |
| E/e’ ratio (per) | 1.009 | 0.934 – 1.090 | 0.822 |  |  |  |  |  |  |  |  |
| Induction of sustained non-  CTI-dependent AFL/other focal AT or  multiple focal AT (yes) | 12.131 | 1.467 – 100.339 | 0.021 |  | 15.745 | 1.610 – 153.944 | 0.018 |  | 13.549 | 1.545 – 118.794 | 0.019 |

AF = atrial fibrillation; AFL = atrial flutter; AT = atrial tachycardia; ATTRwt-CM = wild-type transthyretin amyloid cardiomyopathy; BNP = brain natriuretic peptide; CI = confidence interval; eGFR = estimated glomerular filtration ratio; HR = Hazard ratio; hs-cTnT = high-sensitivity cardiac troponin T; IVSTd = interventricular septal wall thickness at end-diastole; LADs = left atrial end-systolic diameter; LVDd = left ventricular end-diastolic diameter; LVDs = left ventricular end-systolic diameter; LVEF = left ventricular ejection fraction; MRI-ECV = extracellular volume fraction in magnetic resonance imaging; PLVWd = posterior left ventricular wall thickness at end-diastole.
