# Supplementary material for: Clinical outcomes of catheter ablation for atrial fibrillation, atrial flutter, and atrial tachycardia in wild-type transthyretin amyloid cardiomyopathy: a proposed treatment strategy for catheter ablation in each arrhythmia
Source: Europace. 2024 Jun 27;26(6):euae155. doi: 10.1093/europace/euae155 (PMC11208780; doi:10.1093/europace/euae155)
Supplement: euae155_Supplementary_Data [file euae155_supplementary_data.zip › Supplemental Table S2.docx]

**Supplemental Table S2. Univariate and multivariate cox regression analysis for recurrence of AT in patients with ATTRwt-CM**

|  | Univariate Cox Regression | | |  | Multivariate Cox Regression | | |  | Multivariate Cox Regression | | |
| --- | --- | --- | --- | --- | --- | --- | --- | --- | --- | --- | --- |
| Variable | HR | 95% Cl | *p* value |  | HR | 95% Cl | *p* value |  | HR | 95% Cl | *p* value |
| Age (per years) | 1.103 | 0.983 – 1.239 | 0.096 |  |  |  |  |  |  | – |  |
| Gender, Male (yes) | 0.587 | 0.121 – 2.849 | 0.508 |  |  |  |  |  |  |  |  |
| MRI-ECV (per %) | 1.038 | 0.953 – 1.129 | 0.393 |  |  |  |  |  |  |  |  |
| Native T1 (per msec) | 0.992 | 0.977 – 1.007 | 0.298 |  |  |  |  |  |  |  |  |
| Persistent form of AT | 6.726 | 0.824 – 54.910 | 0.075 |  |  | – |  |  | 24.571 | 1.877 – 321.712 | 0.015 |
| hs-cTnT (per ng/dL) | 1.012 | 1.000 – 1.025 | 0.049 |  | 1.004 | 0.989 – 1.019 | 0.585 |  |  | – |  |
| eGFR (per ml/min/1.73m^2^) | 0.966 | 0.911 – 1.023 | 0.237 |  |  |  |  |  |  |  |  |
| BNP (per pg/mL) | 1.001 | 0.998 – 1.004 | 0.424 |  |  |  |  |  |  |  |  |
| LVDd (per mm) | 1.004 | 0.881 – 1.143 | 0.958 |  |  |  |  |  |  |  |  |
| LVDs (per mm) | 1.180 | 1.002 – 1.389 | 0.047 |  |  | – |  |  |  | – |  |
| IVSTd (per mm) | 0.844 | 0.607 – 1.174 | 0.314 |  |  |  |  |  |  |  |  |
| PLVWd (per mm) | 0.942 | 0.751 – 1.183 | 0.609 |  |  |  |  |  |  |  |  |
| LVEF (per %) | 0.915 | 0.851 – 0.984 | 0.017 |  | 0.927 | 0.851 – 1.009 | 0.080 |  |  | – |  |
| LADs (per mm) | 0.955 | 0.864 – 1.055 | 0.362 |  |  |  |  |  |  |  |  |
| E/e’ ratio (per) | 1.038 | 0.919 – 1.173 | 0.544 |  |  |  |  |  |  |  |  |
| Multiple focal ATs (yes) | 3.841 | 0.959 – 15.387 | 0.057 |  |  | – |  |  | 18.926 | 1.699 – 210.793 | 0.017 |

abbreviations as in Supplementary Table S1.
