# Supplementary material for: Clinical outcomes of catheter ablation for atrial fibrillation, atrial flutter, and atrial tachycardia in wild-type transthyretin amyloid cardiomyopathy: a proposed treatment strategy for catheter ablation in each arrhythmia
Source: Europace. 2024 Jun 27;26(6):euae155. doi: 10.1093/europace/euae155 (PMC11208780; doi:10.1093/europace/euae155)
Supplement: euae155_Supplementary_Data [file euae155_supplementary_data.zip › Supplementary material.docx]

Supplementary Material

Supplemental Methods

**Catheter Ablation indication**

Because antiarrhythmic drugs are difficult to use due to negative inotropic effect in ATTRwt-CM patients as described in the manuscript, catheter ablation was performed as a Class IIa indication for patients with symptomatic paroxysmal AF and persistent AF prior to initiation of antiarrhythmic therapy with a Class I or III antiarrhythmic medication (2017 HRS/EHRA/ECAS/APHRS/SOLAECE expert consensus statement on catheter and surgical ablation of atrial fibrillation, and JCS/JHRS 2019 Guideline on Non-Pharmacotherapy of Cardiac Arrhythmias). For patients with heart failure, catheter ablation was performed as a Class IIa indication (2017 HRS/EHRA/ECAS/APHRS/SOLAECE expert consensus statement on catheter and surgical ablation of atrial fibrillation, and JCS/JHRS 2019 Guideline on Non-Pharmacotherapy of Cardiac Arrhythmias). In contrast, catheter ablation has a Class IIb indication for asymptomatic AF and was not performed for asymptomatic long-standing persistent AF lasting more than one year (2017 HRS/EHRA/ECAS/APHRS/SOLAECE expert consensus statement on catheter and surgical ablation of atrial fibrillation, and JCS/JHRS 2019 Guideline on Non-Pharmacotherapy of Cardiac Arrhythmias). However, for asymptomatic AF within 1 year of onset, catheter ablation also has a Class IIb indication in the guidelines (2017 HRS/EHRA/ECAS/APHRS/SOLAECE expert consensus statement on catheter and surgical ablation of atrial fibrillation, and JCS/JHRS 2019 Guideline on Non-Pharmacotherapy of Cardiac Arrhythmias), but was recommended because it has been reported that the effectiveness of ablation therapy is comparable to that of symptomatic AF (Int J Cardiol 2013; 168: 3968-3970. [REF#16 in the manuscript]), and that improvement in exercise tolerance, B-type natriuretic peptide (BNP), and quality of life can be seen after treatment, even in asymptomatic AF patients (J Cardiovasc Electrophysiol 2014; 25: 1057-1064. [REF#17 in the manuscript], and Circ J. 2017;81:444-9. [REF#18 in the manuscript]).

Furthermore, Catheter ablation was performed as a Class I indication in patients with symptomatic focal AT as an alternative to pharmacological therapy (2015 ACC/AHA/HRS Guideline for the Management of Adult Patients With Supraventricular Tachycardia). In addition, catheter ablation of the CTI was performed in patients with CTI-dependent atrial flutter that is either symptomatic or refractory to pharmacological rate control (Class I) (2015 ACC/AHA/HRS Guideline for the Management of Adult Patients With Supraventricular Tachycardia). Catheter ablation was also performed in patients with recurrent symptomatic non-CTI-dependent flutter as primary therapy, before therapeutic trials of antiarrhythmic drugs, after carefully weighing potential risks and benefits of treatment options (Class IIa) (2015 ACC/AHA/HRS Guideline for the Management of Adult Patients With Supraventricular Tachycardia). In addition, catheter ablation was considered to perform for asymptomatic patients with recurrent atrial flutter (Class IIb) (2015 ACC/AHA/HRS Guideline for the Management of Adult Patients With Supraventricular Tachycardia).

Specific criteria were not established just because the patient had ATTRwt-CM, and the process for determining the indication for catheter ablation in ATTRwt-CM was the same as the process for determining the indication for catheter ablation in usual cases other than ATTRwt-CM, and these results can be generalized to any institution.

2017 HRS/EHRA/ECAS/APHRS/SOLAECE expert consensus statement on catheter and surgical ablation of atrial fibrillation

| Arrhythmia | Recommendation | Class |
| --- | --- | --- |
| Symptomatic AF prior to initiation of antiarrhythmic therapy with a Class I or III antiarrhythmic medication | Paroxysmal: Catheter ablation is reasonable. | Class IIa |
|  | Persistent: Catheter ablation is reasonable. | Class IIa |
| AF with Congestive heart failure | It is reasonable to use similar indications for AF ablation in selected patients with heart failure as in patients without heart failure. | Class IIa |
| Asymptomatic AF | Paroxysmal: Catheter ablation may be considered in select patients. | Class IIb |
|  | Persistent: Catheter ablation may be considered in select patients. | Class IIb |

2015 ACC/AHA/HRS Guideline for the Management of Adult Patients With Supraventricular Tachycardia

| Arrhythmia | Recommendation | Class |
| --- | --- | --- |
| Focal Atrial Tachycardia | Catheter ablation is recommended in patients with symptomatic focal AT as an alternative to pharmacological therapy. | Class I |
| Atrial Flutter | Catheter ablation of the CTI is useful in patients with CTI-dependent atrial flutter that is either symptomatic or refractory to pharmacological rate control. | Class I |
|  | Catheter ablation is reasonable in patients with recurrent symptomatic non–CTI-dependent flutter as primary therapy, before therapeutic trials of antiarrhythmic drugs, after carefully weighing potential risks and benefits of treatment options. | Class IIa |
|  | Catheter ablation may be reasonable for asymptomatic patients with recurrent atrial flutter. | Class IIb |

JCS/JHRS 2019 Guideline on Non-Pharmacotherapy of Cardiac Arrhythmias

| Arrhythmia | Recommendation | Class |
| --- | --- | --- |
| Atrial Fibrillation | For patients with symptomatic recurrent paroxysmal AF before the initiation of AADs, catheter ablation should be considered as the first-line therapy | Class IIa |
|  | For selected patients with heart failure, it should be considered to use similar indications for AF ablation as in patients without heart failure | Class IIa |
|  | For patients with symptomatic persistent AF, catheter ablation should be considered | Class IIa |
|  | For patients with asymptomatic recurrent paroxysmal AF, catheter ablation may be considered | Class IIb |
|  | For patients with asymptomatic persistent AF, catheter ablation may be considered | Class IIb |
| Focal Atrial Tachycardia | For patients with symptomatic recurrent focal atrial tachycardia, catheter ablation is recommended | Class I |
|  | For patients with recurrent focal atrial tachycardia, especially if incessant, catheter ablation is recommended | Class I |
|  | For patients with focal atrial tachycardia and tachycardiomyopathy, catheter ablation is recommended | Class I |
|  | For patients with focal atrial tachycardia and depressed LV function due to structural heart disease, catheter ablation should be considered | Class IIa |
|  | For patients with symptomatic focal atrial tachycardia who do not prefer antiarrhythmic medication, catheter ablation should be considered | Class IIa |
| Atrial Flutter | Catheter ablation is recommended for patients with typical AFL that is either symptomatic or refractory  to pharmacological rate/rhythm control | Class I |
|  | Catheter ablation is recommended for patients with typical AFL that is either symptomatic or refractory  to pharmacological rate/rhythm control | Class I |
|  | Catheter ablation should be considered for patients with asymptomatic typical AFL, structural heart  disease, and reduced cardiac function | Class IIa |
|  | Catheter ablation of typical AFL should be considered for patients with typical AFL that is induced inadvertently at the time of catheter ablation for tachycardia other than AF | Class IIa |
|  | Catheter ablation should be considered for patients with typical AFL that requires pharmacological  rate/rhythm control therapy | Class IIa |
|  | Catheter ablation may be considered for patients with asymptomatic recurrent typical AFL | Class IIb |

**Study outcomes (follow-up period)**

The Kaplan-Meier analysis showed a follow-up period of 5 years in this study. In recent randomized controlled trials comparing catheter ablation with medical therapy for AF complicated by heart failure, with a primary endpoint of reduction in all-cause mortality or heart failure, the follow-up period was 37.6 months in the CASTLE-AF trial (N Engl J Med. 2018;378:417-427.), 49 months in the CABANA trial (Circulation. 2021;143:1377-1390.), and 37 months in the RAFT-AF trial (Circulation. 2022;145:1693-1704.). Therefore, a minimum follow-up period of 3 years was considered necessary to evaluate adequately the improvement in prognosis and heart failure hospitalizations after catheter ablation for AF/AFL/AT complicated by ATTRwt-CM.

Furthermore, there are some studies with 5-year long-term follow-up on the outcome of catheter ablation for long-standing persistent AF (Circ Arrhythm Electrophysiol. 2015;8:18-24., J Am Coll Cardiol. 2012;60:1921-1929.), and especially since late recurrence can occur 12 months or more after AF ablation, a longer follow-up study was thought to possibly provide some insight.

In addition, since the ATTR-ACT trial (N Engl J Med. 2018;379:1007-1016.), in which the median age was 75 years, reported that tafamidis improved the prognosis of patients with ATTRwt-CM, with a survival rate of 70.5% at 30 months in the tafamidis group, the prognosis of ATTRwt-CM patients is expected to be 5 years or longer.

Therefore, it is important to show the long-term results of catheter ablation in patients with ATTRwt-CM, and Kaplan-Meier analysis was performed with a follow-up period of 5 years.

**Statistical analysis**

The distribution of continuous data was evaluated using the Shapiro–Wilk test. Continuous variables with normal distributions were expressed as mean ± standard deviation, and non-normally distributed variables were presented as median values with an interquartile range. Comparisons of continuous variables between groups were performed by one-way analysis of variance using Tukey’s t-test for normally distributed variables or the Kruskal–Wallis test for non-normally distributed variables. Categorical variables are expressed as frequencies and percentages and compared using the χ^2^ test. A multivariate Cox regression analysis was performed to adjust for the influence of other factors and determine the impact of arrhythmia or CA on mortality and hospitalization due to HF. Univariate and multivariate logistic and Cox regression analyses were performed to identify significant parameters related to the occurrence or recurrence of each arrhythmia. If a correlation was detected between parameters, the multivariate analysis was performed in multiple models, leaving one of the factors with internal correlations. The clinical course of mortality, HF hospitalization, and recurrence of each arrhythmia was demonstrated using the Kaplan–Meier analysis. All analyses were performed using SPSS version 25 software (SPSS Inc., Chicago, IL, USA). All statistical tests were two-sided, and a p-value <0.05 was regarded as statistically significant.

Supplementary Discussion

of the results of previous reports and the present study

There are a few small case reports detailing CA for AF/AFL/AT complicated by cardiac amyloidosis. Black-Maier et al. reported that 10 patients with amyloidosis (one amyloid light-chain [AL], nine ATTRwt) underwent CA for AF/CTI-dependent AFL, of whom seven had persistent AF, and that six (60%) had recurrence after 1 year and eight (80%) after 2 years.^23^ Among them, six patients who had recurrence at 1 year were relatively elderly compared to four patients without recurrence, indicating that the treatment outcome of persistent AF in elderly patients might be poor, while ablation might be effective in younger patients considered non-progressive. However, in the present study, age also tended to be associated with recurrence of AF/AFL/AT in persistent AF patients, at HR: 1.100 (95% CI: 0.964–1.254; p=0.155) in univariate analysis, but not significantly (Supplemental Table S1), suggesting that age alone may not be a determinant of recurrence. On the other hand, Donnellan E et al. reported that CA was performed in 24 amyloidosis patients (21 ATTRwt, three hereditary ATTR [ATTRv]) with AF (20 persistent, four paroxysmal) and that only one of 10 patients with UK National Amyloidosis Centre stage 3 (NT-proBNP >3,000 ng/mL, eGFR <45 mL/min/m^2^) (Eur Heart J. 2018;39:2799-2806.) had no recurrence for 3 years, while nine of 14 patients with National Amyloidosis Centre stage 1 or 2 were free of recurrence, suggesting the usefulness of CA in patients without evidence of advanced amyloidosis.^24^ Furthermore, compared with 48 matched background amyloidosis patients without CA (36 ATTRwt, 12 ATTRv), the ablation group had fewer hospitalizations due to arrhythmias (1.7±2.4 vs. 4.0±3.5 hospitalizations, p=0.005) and improved prognosis (HR: 0.38, 95% CI: 0.17–0.86, p=0.02), indicating that ablation might even improve prognosis. This study also showed that CA for AF/AFL/AT significantly improved all-cause mortality, cardiovascular mortality, and HF hospitalization in multivariate Cox regression and Kaplan–Meier analyses compared with no CA. Furthermore, higher hs-cTnT levels and induction of non-CTI-dependent AFL/focal AT were independent predictors of AF/AFL/AT recurrence in patients with persistent AF (Supplemental Table S1), and higher MRI-ECV (especially over 61.1%) is independently associated with the occurrence of non-CTI-dependent complex AFL or multiple focal AT (Table 8, and Figure 6), indicating that complex AFL/multiple focal AT is more likely to occur in advanced cases and may be expected to have a poor clinical outcome.

On the other hand, Barbhaiya CR et al. showed that in 18 amyloidosis patients (four AL, 14 ATTRwt) with persistent AF, voltage maps were performed in seven patients and showed that the left atrial voltage was lower than that in non-amyloidosis patients matched for age and sex and that AT was induced more frequently (3.3±1.9 ATs vs 0.2±0.4 ATs, p<0.001) and the recurrence rate at 1 year was 83% compared to 25% in the non-amyloidosis group with an HR of 5.4, which is extremely poor.^25^ Although the present study was not able to examine the level of atrial voltage in all cases, there is agreement that many cases had somewhat low-voltage areas and that AT is often present in 18 of the 54 patients (33%; Figure 2), which is consistent with the previous study. Furthermore, although the treatment outcome was not very poor in the present study, it would have been extremely poor if complex AFLs or multiple focal ATs were observed; thus, there might have been many complex AFLs and multiple focal ATs in the study by Barbhaiya et al. Finally, Tan NY et al. reported that 13 patients with amyloidosis (five AL, seven ATTRwt, and one ATTRv) underwent ablation in three patients with AF, six with CTI-dependent AFL, two with AF and CTI-dependent AFL, and two with AT, and that the recurrence-free rate was 75% at 1 year and 60% at 3 years, which was not good by any means, but another 13 patients (two AL, 10 ATTRwt, and one ATTRv) underwent AV node ablation and reported similar NYHA improvement compared to the ablation group.^26^ AV node ablation may be an optional strategy in advanced cases of amyloidosis.
