# Supplementary figures and images for: Clinical outcomes of catheter ablation for atrial fibrillation, atrial flutter, and atrial tachycardia in wild-type transthyretin amyloid cardiomyopathy: a proposed treatment strategy for catheter ablation in each arrhythmia
Source: Europace. 2024 Jun 27;26(6):euae155. doi: 10.1093/europace/euae155 (PMC11208780; doi:10.1093/europace/euae155)

## Slide 1
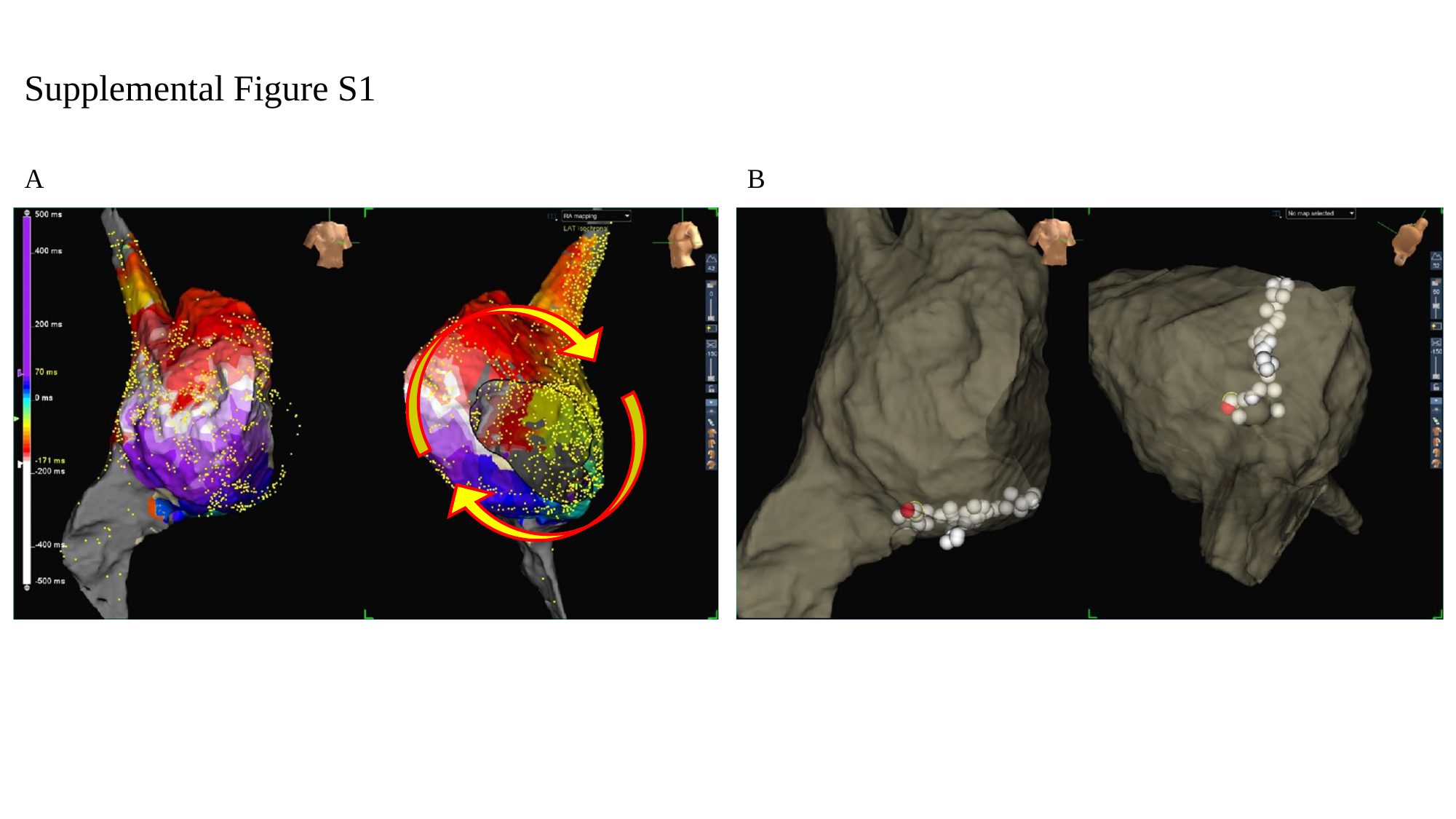

Supplemental Figure S1
A
B

Supplement: euae155_Supplementary_Data [file euae155_supplementary_data.zip › Supplemental Figure S1.pptx]

## Slide 1
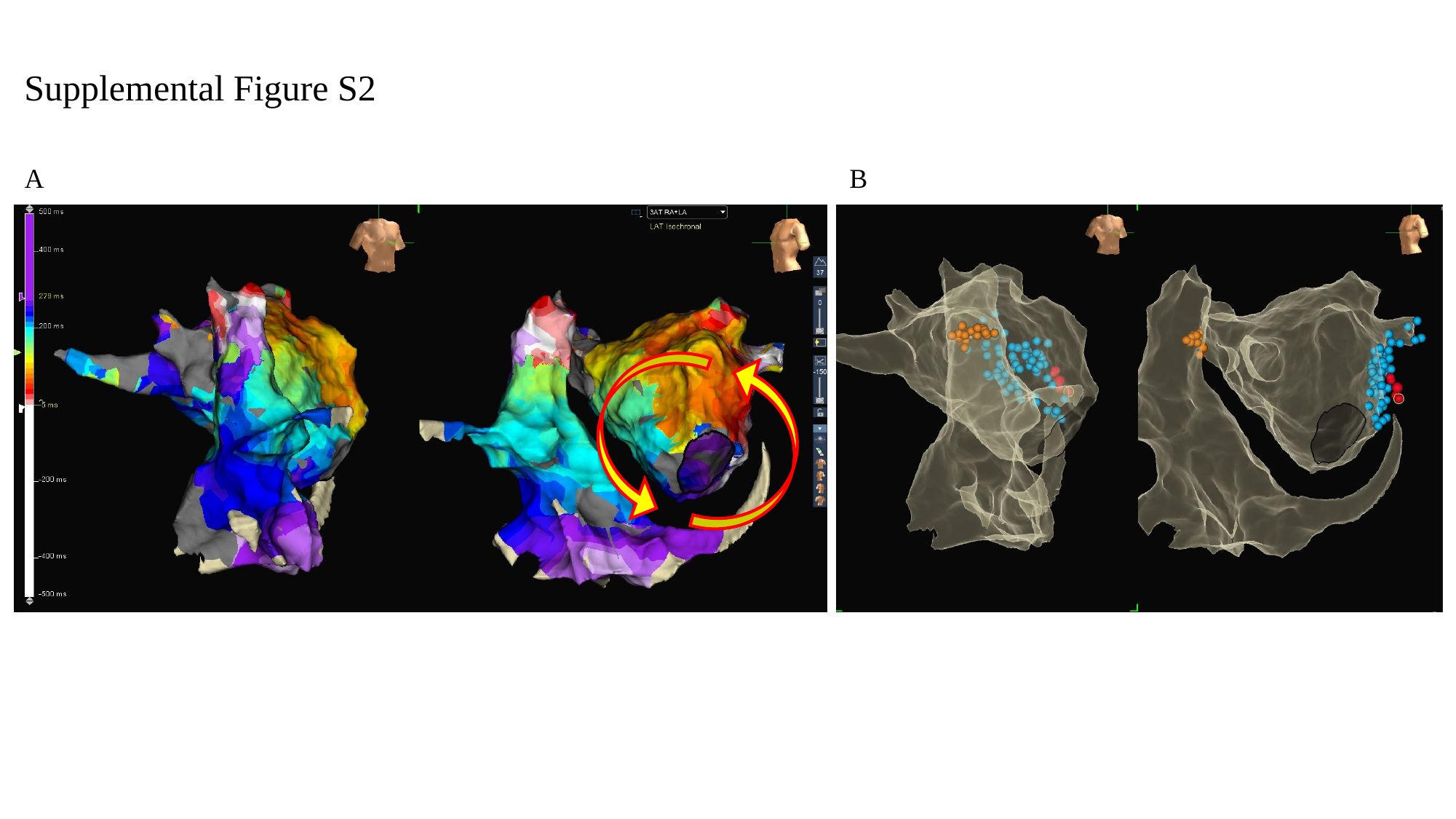

Supplemental Figure S2
A
B

Supplement: euae155_Supplementary_Data [file euae155_supplementary_data.zip › Supplemental Figure S2.pptx]

## Slide 1
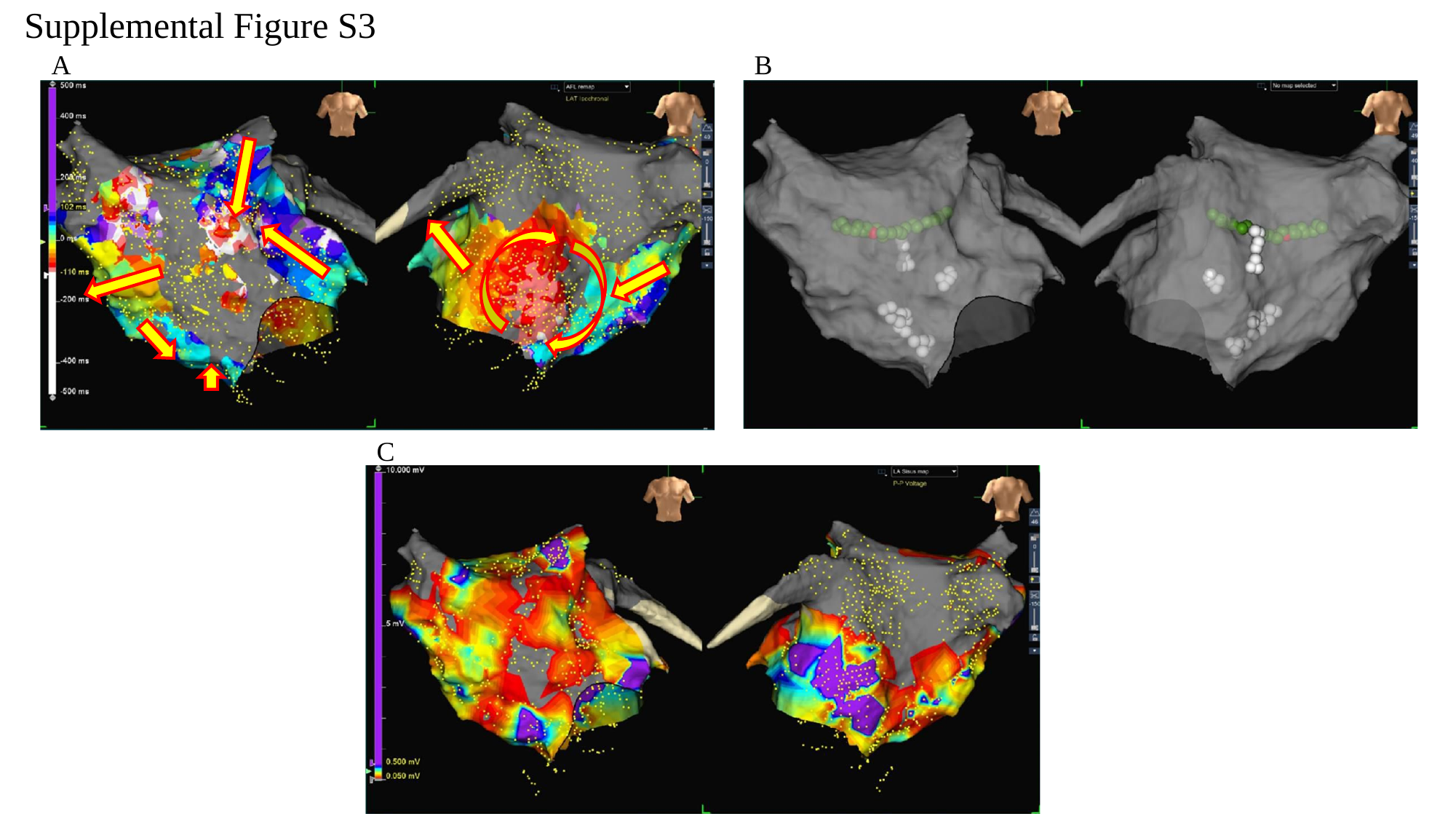

Supplemental Figure S3
A
B
C

Supplement: euae155_Supplementary_Data [file euae155_supplementary_data.zip › Supplemental Figure S3.pptx]

## Slide 1
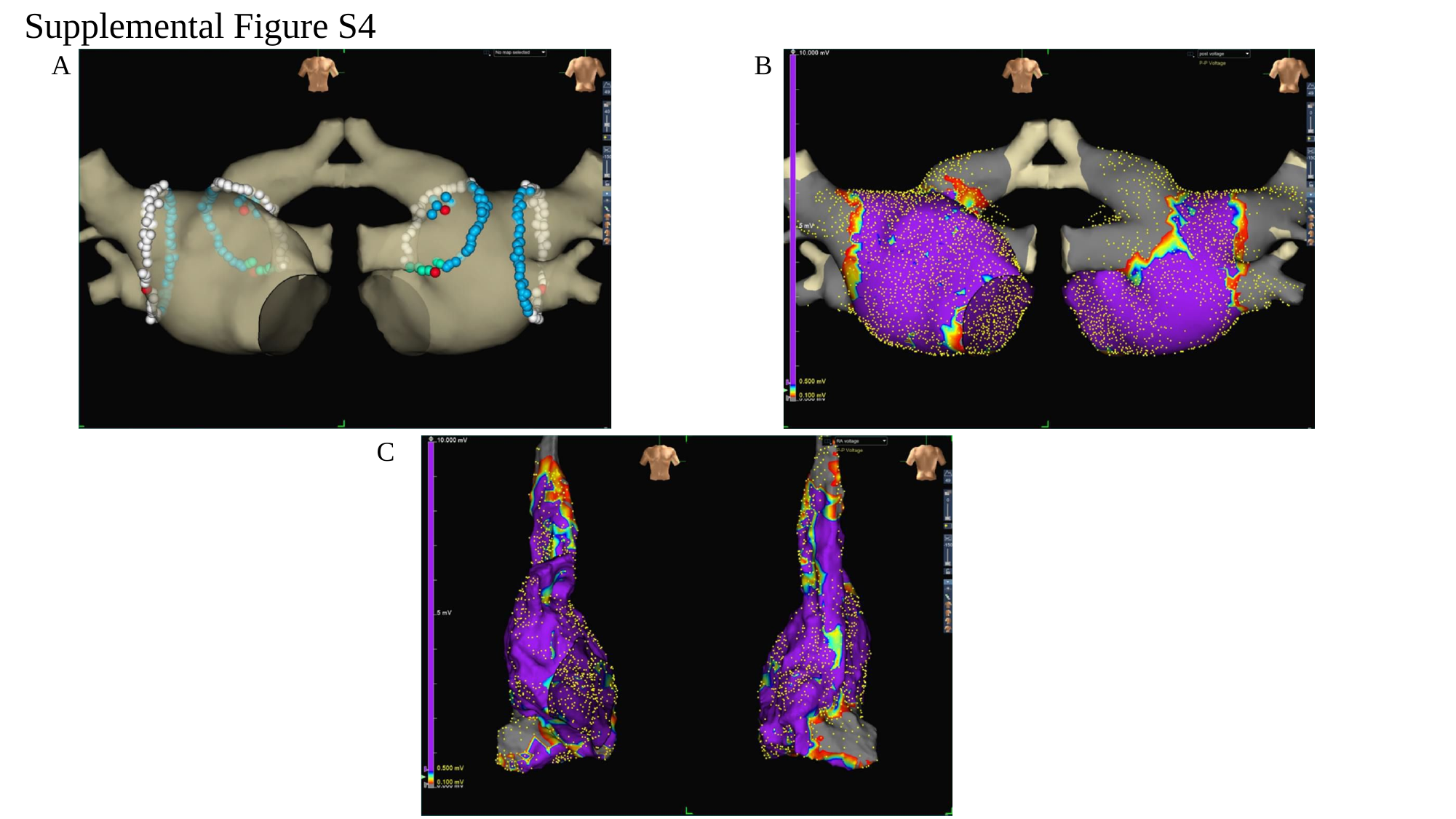

Supplemental Figure S4
A
B
C

Supplement: euae155_Supplementary_Data [file euae155_supplementary_data.zip › Supplemental Figure S4.pptx]

## Slide 1
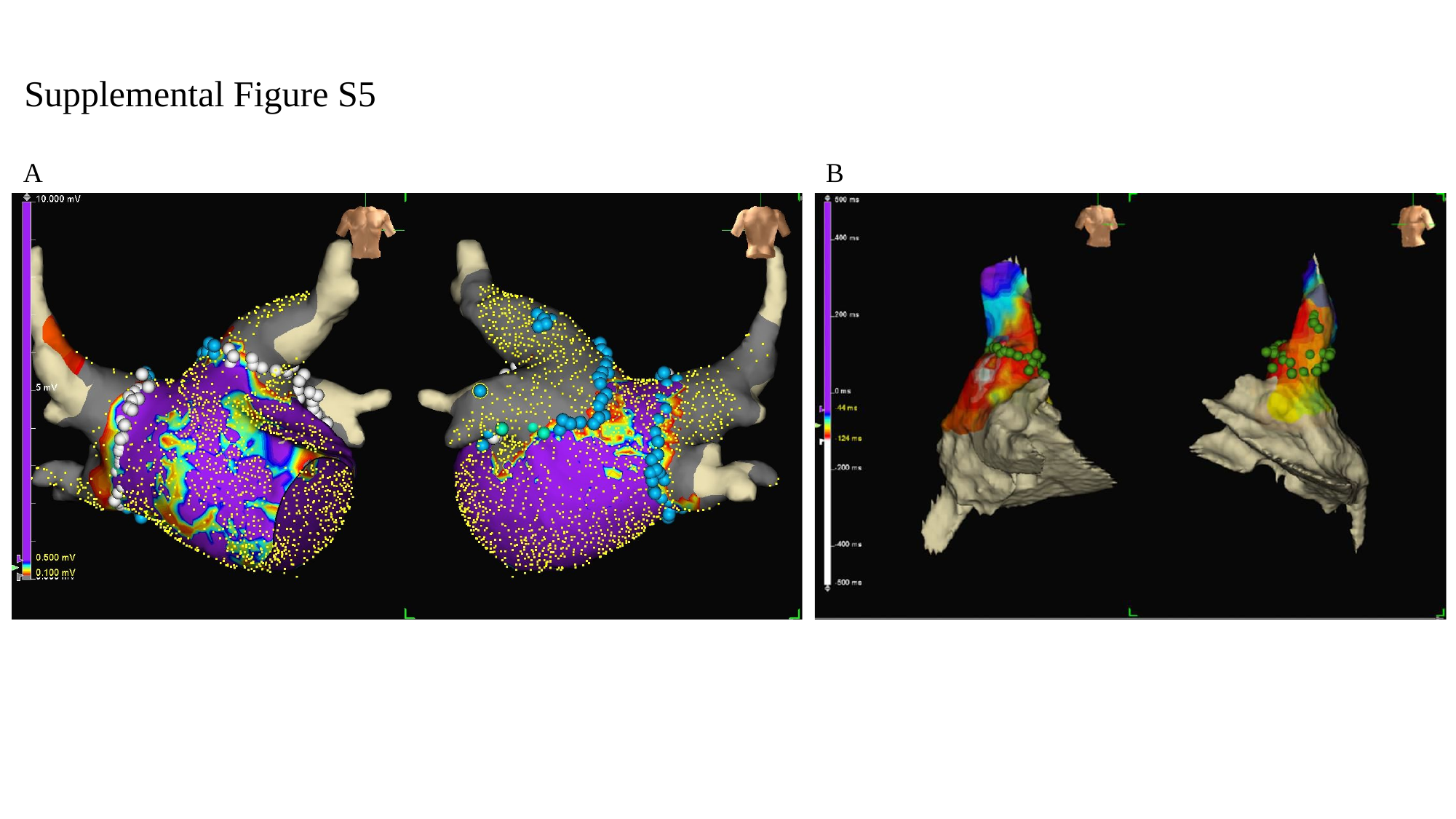

Supplemental Figure S5
A
B
C

Supplement: euae155_Supplementary_Data [file euae155_supplementary_data.zip › Supplemental Figure S5.pptx]

## Slide 1
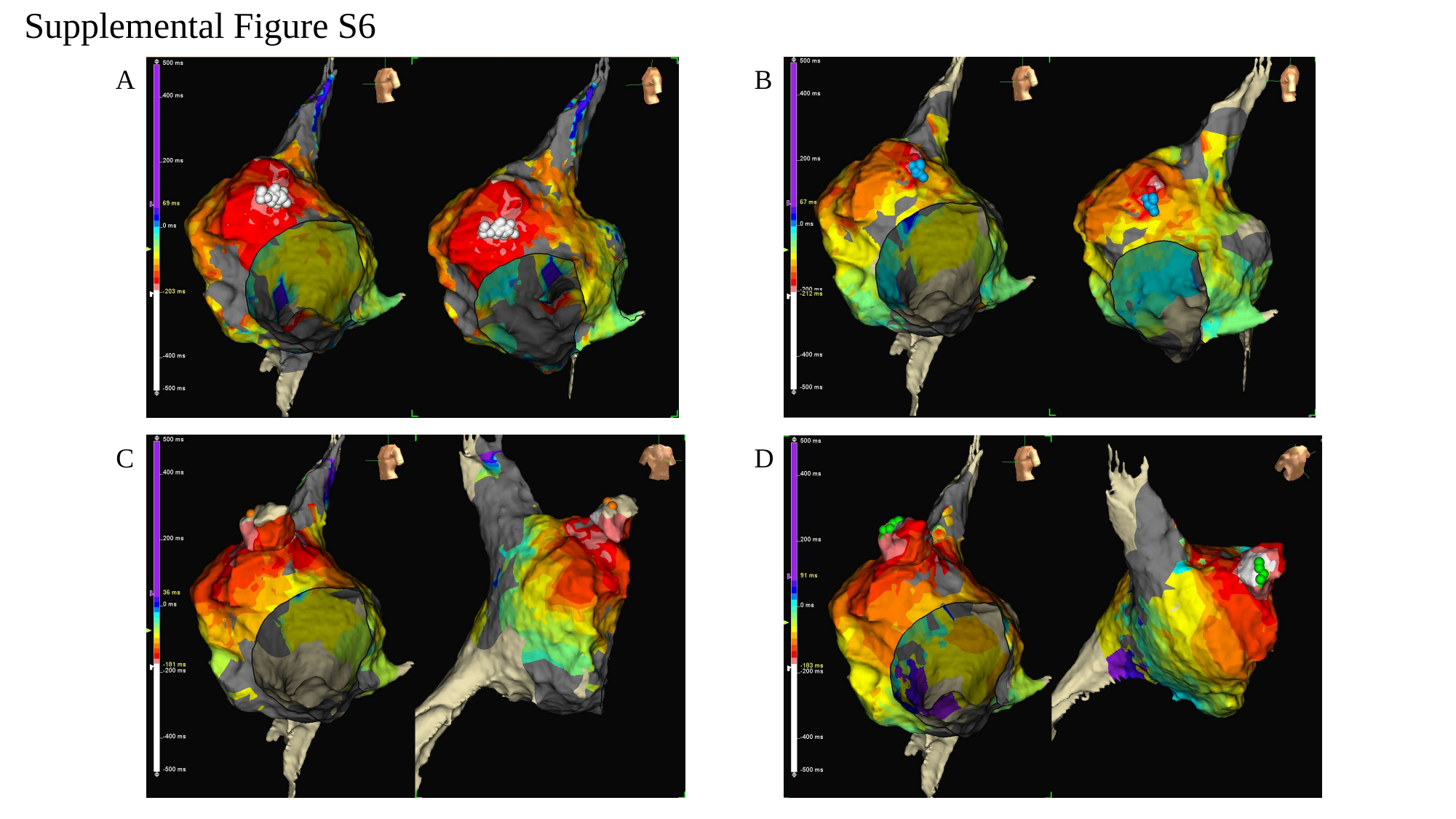

Supplemental Figure S6
A
B
C
D

Supplement: euae155_Supplementary_Data [file euae155_supplementary_data.zip › Supplemental Figure S6.pptx]
